# Enhancement of target specificity of CRISPR–Cas12a by using a chimeric DNA–RNA guide

**DOI:** 10.1093/nar/gkaa605

**Published:** 2020-07-20

**Authors:** Hanseop Kim, Wi-jae Lee, Yeounsun Oh, Seung-Hun Kang, Junho K Hur, Hyomin Lee, WooJeung Song, Kyung-Seob Lim, Young-Ho Park, Bong-Seok Song, Yeung Bae Jin, Bong-Hyun Jun, Cheulhee Jung, Dong-Seok Lee, Sun-Uk Kim, Seung Hwan Lee

**Affiliations:** Futuristic Animal Resource & Research Center (FARRC), Korea Research Institute of Bioscience and Biotechnology (KRIBB), Cheongju, Korea; School of Life Sciences and Biotechnology, BK21 Plus KNU Creative BioResearch Group, Kyungpook National University, Daegu, Republic of Korea; Futuristic Animal Resource & Research Center (FARRC), Korea Research Institute of Bioscience and Biotechnology (KRIBB), Cheongju, Korea; Department of Bioscience and Biotechnology, Konkuk University, Seoul 143-701, Republic of Korea; Futuristic Animal Resource & Research Center (FARRC), Korea Research Institute of Bioscience and Biotechnology (KRIBB), Cheongju, Korea; Department of Biotechnology, College of Life Sciences and Biotechnology, Korea University, Seoul 02841, Republic of Korea; Futuristic Animal Resource & Research Center (FARRC), Korea Research Institute of Bioscience and Biotechnology (KRIBB), Cheongju, Korea; Department of Medicine, Graduate School, Kyung Hee University, Seoul 02447, Republic of Korea; Department of Pathology, College of Medicine, Kyung Hee University, Seoul 02447, Republic of Korea; Department of Biomedical Science, Graduate School, Kyung Hee University, Seoul 02447, Republic of Korea; Department of Medical Genetics, College of Medicine, Hanyang University, Seoul, Republic of Korea; Department of Biomedical Science, Graduate School, Kyung Hee University, Seoul 02447, Republic of Korea; Department of Biomedical Science, Graduate School, Kyung Hee University, Seoul 02447, Republic of Korea; Futuristic Animal Resource & Research Center (FARRC), Korea Research Institute of Bioscience and Biotechnology (KRIBB), Cheongju, Korea; Futuristic Animal Resource & Research Center (FARRC), Korea Research Institute of Bioscience and Biotechnology (KRIBB), Cheongju, Korea; Futuristic Animal Resource & Research Center (FARRC), Korea Research Institute of Bioscience and Biotechnology (KRIBB), Cheongju, Korea; National Primate Research Center (NPRC), Korea Research Institute of Bioscience and Biotechnology (KRIBB), Cheongju, Republic of Korea; Department of Bioscience and Biotechnology, Konkuk University, Seoul 143-701, Republic of Korea; Department of Biotechnology, College of Life Sciences and Biotechnology, Korea University, Seoul 02841, Republic of Korea; School of Life Sciences and Biotechnology, BK21 Plus KNU Creative BioResearch Group, Kyungpook National University, Daegu, Republic of Korea; Futuristic Animal Resource & Research Center (FARRC), Korea Research Institute of Bioscience and Biotechnology (KRIBB), Cheongju, Korea; Department of Functional Genomics, KRIBB School of Bioscience, Korea University of Science and Technology (UST), Daejeon, Republic of Korea; National Primate Research Center (NPRC), Korea Research Institute of Bioscience and Biotechnology (KRIBB), Cheongju, Republic of Korea

## Abstract

The CRISPR–Cas9 system is widely used for target-specific genome engineering. CRISPR–Cas12a (Cpf1) is one of the CRISPR effectors that controls target genes by recognizing thymine-rich protospacer adjacent motif (PAM) sequences. Cas12a has a higher sensitivity to mismatches in the guide RNA than does Cas9; therefore, off-target sequence recognition and cleavage are lower. However, it tolerates mismatches in regions distant from the PAM sequence (TTTN or TTN) in the protospacer, and off-target cleavage issues may become more problematic when Cas12a activity is improved for therapeutic purposes. Therefore, we investigated off-target cleavage by Cas12a and modified the Cas12a (cr)RNA to address the off-target cleavage issue. We developed a CRISPR–Cas12a that can induce mutations in target DNA sequences in a highly specific and effective manner by partially substituting the (cr)RNA with DNA to change the energy potential of base pairing to the target DNA. A model to explain how chimeric (cr)RNA guided CRISPR–Cas12a and SpCas9 nickase effectively work in the intracellular genome is suggested. Chimeric guide-based CRISPR- Cas12a genome editing with reduced off-target cleavage, and the resultant, increased safety has potential for therapeutic applications in incurable diseases caused by genetic mutations.

## INTRODUCTION

The CRISPR–Cas system is a bacterial immune system, which and it is now widely used for target-specific genome editing in various organisms (1). One of the CRISPR systems, CRISPR–Cas9 RNA-guided endonuclease, is routinely used to specifically correct or control genes of interest based on its ability to cut double-stranded DNA (1–5). Another component of the CRISPR system, CRISPR–Cas12a (Cpf1) was recently reported and is a class II, type V effector nuclease that has a bi-lobed structure composed of nuclease and recognition domains, similar to Cas9 (6,7). Cas12a binds the target DNA helix via a single-stranded (cr)RNA, forming a DNA–RNA hybrid duplex (6,8). Cas12a has attracted attention as an excellent genome-engineering tool as it overcomes certain limitations of Cas9 (9–12). In contrast to Cas9, which recognizes guanine (G)-rich sequences, Cas12a recognizes specific thymine (T)-rich protospacer adjacent motif (PAM) sequences (TTTN or TTN) and can specifically induce double-strand DNA cleavage (7,13). The CRISPR–Cas12a is now broadly applicable for gene editing in various organisms ranging from microorganisms to humans, and therefore, many researchers have tried to engineer the CRISPR– Cas12a protein or (cr)RNA for specific purposes (14–17). In particular, for safety issues, (cr)RNAs, which are more accessible than proteins, can be engineered to improve the genome-editing specificity. Cas12a recognizes a 24 base protospacer sequence, which is composed of a 20 base pair long guide-target heteroduplex and the last four nucleotides, which is separated from the guide RNA. It is known that 5–10 base distances from the PAM within the protospacers are important for DNA target recognition as a seed region (18). CRISPR–Cas12a is more sensitive to mismatches between the target DNA and the gRNA than CRISPR–Cas9; when a mismatch is introduced into the seed sequence in the protospacer, its cleavage activity is significantly inhibited (19,20). However, there is still a possibility of mismatch cleavage in regions other than the seed region, which can currently not be detected, and therefore, the off-target cleavage issue is not entirely resolved. Substitution of the (cr)RNA with DNA possibly increases the target specificity by changing the binding energy between the guide and target (21). In addition, DNA is more stable than RNA in aqueous solution and thus is more easily applicable in genome editing and more convenient for product commercialization. Therefore, in the current study, we partially replaced the gRNA for CRISPR–Cas12a with DNA in an attempt to improve the accuracy of the system and to diversify the conventional CRISPR–Cas12a genome editing tool. Chimeric DNA–RNA guides with high target specificity were screened by measuring the cleavage efficiency of each chimeric guide–Cas12a complex based on different on- and off-target DNA sequences. We thus identified a chimeric guide with high accuracy that unperturbed on-target cleavage activity. This novel system is advantageous in terms of safety and has application potential for various purposes *in vivo* (22,23), and will eventually be useful for gene therapy for diseases caused by genetic defects.

## MATERIALS AND METHODS

### Preparation of the CRISPR–Cas12a recombinant protein and chimeric guides

For purification of Cas12a recombinant protein, pET28a–Cas12a (*Acidaminococcus* sp. (As) Cas12a, *Lachnospiraceae bacterium* (Lb) Cas12a, *Francisella novicida* (Fn) Cas12a) bacterial expression vectors were introduced into *Escherichia coli* BL21 (DE3) species and transformed, and then cultured at 37°C until the O.D. reached 0.6. After 48 h of IPTG inoculation, bacterial cells were precipitated to remove the culture medium, and the remaining cell pellet was resuspended in lysis buffer [20 mM Tris–HCl (pH8.0), 300 mM NaCl, 10 mM β-mercaptoethanol, 1% TritonX-100, 1 mM PMSF]. Then, bacterial cell membranes were broken by sonication (ice, 3 min), and the cell lysate was harvested by centrifugation (5000 rpm, 10 min). For single-step (using 6xHis at N-terminus of Cas12a) purification, Ni-NTA resin pre-washed with wash buffer [20 mM Tris–HCl (pH8.0), 300 nM NaCl] and the ultrasonically disrupted intracellular solution were mixed and stirred for 1 h 30 min in a cold room (4°C). After bacterial cell precipitation, non-specific binding components were removed by washing with buffer B [20 mM Tris–HCl (pH 8.0), 300 nM NaCl] at 10 times volume, elution buffer [20 mM Tris–HCl (pH 8.0), 300 nM NaCl, 200 mM Imidazole] was used to elute the AsCas12a protein. The buffer which is used for protein elution was exchanged to storage buffer [200 mM NaCl, 50 mM HEPES (pH 7.5), 1 mM DTT, 40% glycerol] using a centricon (Amicon Ultra) and stored at –80°C. For two-step purification, eluted proteins were further incubated with anti-FLAG resin for 1 h 30 min in a cold room (4°C). Non-specific binding components were washed out with wash buffer [20 mM Tris–HCl (pH8.0), 300 nM NaCl] again. After washing, elution buffer [20 mM Tris–HCl (pH 8.0), 300 nM NaCl, 3xFLAG peptide (5 mg/ml)] was used to elute the Cpf12a protein. The eluted buffer is exchanged against to storage buffer [200 mM NaCl, 50 mM HEPES (pH 7.5), 1 mM DTT, 40% glycerol] by using centricon (Amicon Ultra,) and stored at –80°C. Chimeric DNA–RNA guides (bioneer) were batch synthesized according to the target sequences in each target gene (Supplementary Table S1).

### In-vitro transcription and purification of the guide RNA for Cas12a and Cas9

For *in vitro* transcription, each sense and anti-sense DNA oligo containing the target (cr)RNA sequence (Supplementary Table S1) was purchased (Macrogen). The annealed DNA template was mixed with T7 RNA polymerase (NEB) and the reaction mixture (50 mM MgCl_2_, 100 mM rNTP (Jena Bio, NU-1014), 10× RNA polymerase reaction buffer, RNase Inhibitor Murine, 100 mM DTT, DEPC). (cr)RNA was synthesized by incubation for 8 h at 37°C. After synthesis, the DNA template was completely removed by incubation at 37°C for 1 h with DNase, and only the RNA was separated through the column (MP Biomedicals, GENECLEAN^®^ Turbo Kit). Purified RNA was concentrated through lyophilization (2000 rpm, 1 h, −55°C, 25°C).

### *In-vitro* DNA cleavage assay and calculation of the DNA cleavage efficiency

On-/off-target PCR amplicons are obtained from purified genomic DNA (HEK293FT) using DNA primers (Supplementary Table S2) corresponding to each target gene (*DNMT1, HPRT1, RPL32P3, CCR5, FANCF, GRIN2B, EMX1*). To cleave the amplicons, purified recombinant Cas12a protein and synthesized chimeric (cr)RNA–DNA (purchased from Bioneer) or purified (cr)RNA corresponding to each locus were premixed and incubated at 37°C for 1 h in cleavage buffer (NEB3, 10 μl volume). Then, the reaction was stopped by adding a stop buffer (100 mM EDTA, 1.2% SDS). DNA cleavage was checked by 2% agarose gel electrophoresis. DNA cleavage efficiency was determined by calculating the image pattern according to the formula (Intensity of the cleaved fragment/total sum of the fragment intensity × 100 = %) measured using ImageJ software(NIH).

### Cell sub-culture and transfection

HEK293FT cell line (ATCC) was passaged in DMEM media (DMEM (Gibco) with 10% FBS (Gibco)) every 48 h at 37°C, 5% CO_2_ to maintain a confluency of 70%. For efficient endogenous locus editing, we used single transfection method with electroporation (Lonza, V4XC-2032) or sequential transfection with electroporation and lipofection method. For single transfection with electroporation, 10^5^ cells were mixed with Cas12a-chimeric guide pre-mixed complex (Cas12a: 60 pmol, (cr)RNA: 240 pmol) and followed by electric shock (program: CM-130) in electroporation buffer (manufacturer's guide). Subsequently, transfer the transfected cells into pre-incubated (37°C and 5% CO_2_) DMEM media solution (500 ul) of a 24-well plate, and incubate at the same conditions (37°C and 5% CO_2_) for 72 h. In case of the sequential transfection, HEK293FT cells were nucleofected with vector mixture (human codon optimized) for AsCpf1 (500ng), d/n-SpCas9 (D10A, H840A for dead / D10A for nickase, 100 ng), crRNA (150 pmol), and sgRNA (15 pmol) expression. After 12 h, same amount of crRNA and sgRNA were transfected with 2.8 μl of lipofectamine (ThermoFisher) and 2.0 μl of P3000 reagent twice at the same interval. Seventy two hours after transfection, genomic DNA was extracted from HEK293FT cells and analyzed by targeted amplicon sequencing (illumina, SY-420-1001). To induce the mutation on plasmid target sequence, target plasmids (*DNMT1, GRIN2B* on-/off-target plasmid, 1μg) were co-transfected with Cas12a–crRNA pre-mixture and extracted target plasmids were further analyzed by targeted amplicon sequencing (illumina, SY-420-1001).

### Genomic DNA purification

Forty eight hours after the delivery of the chimeric (cr)RNA–DNA and the recombinant Cas12a protein complex into cells (HEK293FT), genomic DNA was isolated using a genomic DNA purification kit (Qiagen, DNeasy Blood & Tissue Kit) according to the manufacturer's protocol. In the case of plasmid delivery, genomic DNAs were extracted after 72 h transfection.

### Targeted amplicon sequencing and data analysis

PCR amplicons (*DNMT1, HPRT1, RPL32P3, CCR5, FANCF, GRIN2B, EMX1*) were prepared by using DNA primer (Supplementary Table S2) corresponding to the target locus. Then, nested PCR (denaturation: 98°C – 30 s, primer annealing: 58°C – 30 s, elongation: 72°C – 30 s, 35 cycles) was performed to insert the adapter and index sequences into both 5′ and 3′-end of the amplicon (denaturation: 98°C – 30 s, primer annealing: 62°C – 15 s, elongation: 72°C – 15 s, 35 cycles). Thereafter, the tagged amplicon mixture was loaded onto a mini-SEQ analyzer (Illumina, SY-420-1001) according to the manufacturer's guidelines and subjected to targeted deep sequencing. The saved Fastq files were analyzed with Cas-Analyzer (24) and the indel efficiency was calculated.

## RESULTS

### Effects of DNA substitution in the 5′,3′-end of the (cr)RNA on Cas12a activity

**Figure 1.**
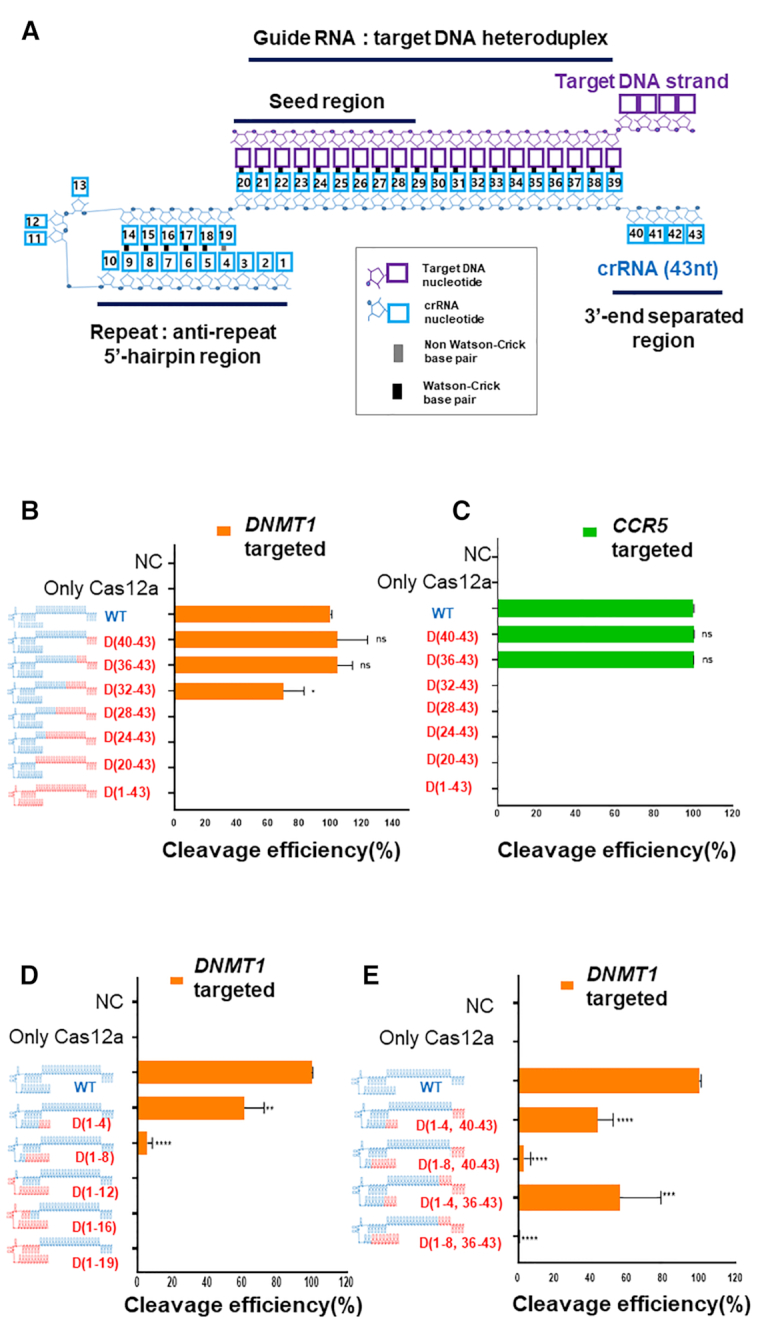
Target DNA cleavage by CRISPR-Cas12a (Cpf1) using chimeric DNA–RNA guides. (**A**) Schematic representation of interactions of AsCas12a (cr)RNA (colored in cyon) with target DNA (colored in purple) (6). The AsCas12a (cr)RNA was numbered from 5′-end to 3′-end. (**B, C**) The target DNA amplicon cleavage efficiency of AsCas12a with partial DNA substitution of (cr)RNA was determined for target sequence in (B) *DNMT1* (orange) and (C) *CCR5* (green), respectively. The (cr)RNA was replaced with DNA from 3′-end with a 4-nt interval. The RNA portion of the (cr)RNA is shown in blue, and the DNA portion is shown in red ('D' indicates a DNA and the position number of substituted DNA nucleotides in (cr)RNA is indicated). The X-axis indicates the efficiency of the target gene (*DNMT1* (orange), *CCR5* (green)) cleavage by AsCas12a using various chimeric DNA–RNA guides (DNA substitution of 4-nt from the 3′-end of the (cr)RNA). Y-axis indicates used chimeric (cr)RNAs in the cleavage experiment. (**D, E**) Target gene (*DNMT1*) cleavage by AsCas12a using chimeric DNA–RNA guides (Serial 4-nt DNA substitution from the 5′-end of the (cr)RNA, (D)) or chimeric DNA–RNA guides (Combination of DNA substitution in the 5′ or 3′-end of the (cr)RNA, (E)). All cleavage efficiency were calculated from agarose gel separated band intensity (cleaved fragment intensity (%)/total fragment intensity (%)) and normalized to wild-type (cr)RNA (Figure S1). Data are shown as means ± s.e.m. from three independent experiments. *P*-values are calculated using a two-tailed Student's *t*-test (ns: not significant, *P< 0.05, **P< 0.01, ***P< 0.001, ****P< 0.0001).

The (cr)RNA and target DNA recognition site of Cas12a have been well characterized (6,8,18,25–27). Cas12a proteins, including *Acidaminococcus* sp. Cas12a (AsCas12a), generally bind with the target DNA to form a target DNA–gRNA duplex in a non-target-sequence-dependent fashion via positively charged amino acids in the REC1, REC2 and RuvC domains. In particular, the seed proximal region (5–10 bp from the PAM) is recognized by the amino acid residues of WED-REC1-RuvC and is essential for target DNA recognition, and it constitutes a protospacer with relatively less sensitive PAM distal regions (6). Cas12a amino acid residues that recognize the 2′-OH group in the (cr)RNA are known (His872, Glu786, Asn175, Arg176, Arg192, Arg518, Asn515, Gly270, Lys273, Gln286). A structurally conserved aromatic residue disrupts guide RNA–target DNA base pairing at the 3′ end of the guide RNA, limiting the duplex to 20 base-pairs. To change the interaction between the (cr)RNA and Cas12a protein, we replaced part of the (cr)RNA sequence in the 5′-side hairpin region, a single-strand region separated by a tryptophan residue (Y382) at the 3′-side and the protospacer DNA–RNA hybrid region, with DNA (Figure 1A). First, we sequentially replaced 4-nt regions of the RNA guide with DNA starting from the 3′ end, the cleavage efficiency for each target (*DNMT1, CCR5*) was compared (Figure 1B, C, Supplementary Figures S1, S2, Supplementary Table S1). All the cleavage assay was conducted using recombinant Cas12a proteins which shows high purity with robust cleavage activity (Supplementary Figure S3). PCR amplicon cleavage of the two target genes revealed that up to 8 nt of DNA substitution starting from the 3′-end did not significantly affect the target cleavage efficiency. However, when more than nine consecutive bases in the guide were substituted, this resulted in a significant decrease in on-target sequence cleavage efficiency, and no cleavage was observed when more than 12 nt were substituted in Cas12a from various sources (Supplementary Figure S4A). This suggests that 2′-OH recognition of the (cr)RNA is largely conserved among Cas12a proteins and that the sensitivity of the 2′-OH recognition by CRISPR–Cas12a increases from the PAM (TTTN or TTN) distal to the PAM proximal region. This is in line with previous findings in experiments in which mismatches were sequentially introduced into the gRNA and target DNA heteroduplex regions (18). When the RNA nucleotides from the 5′-end of the hairpin region other than the protospacer were replaced with DNA, the chimeric DNA–RNA guided Cas12a showed a significant decrease in cleavage efficiency when compared to Cas12a with the wild-type guide (Figure 1D). Similarly, Cas12a activity decreased when the conserved region of the 5′-hairpin structure and 3′-end was replaced simultaneously (Figure 1E). These effects were in agreement with a previous study (7). These findings indicate that the 2′-OH in the 5′- hairpin region, which forms a pseudoknot, is critically required for Cas12a protein recognition of the (cr)RNA.

### Effects of DNA substitution in the seed region of the (cr)RNA on Cas12a activity

**Figure 2.**
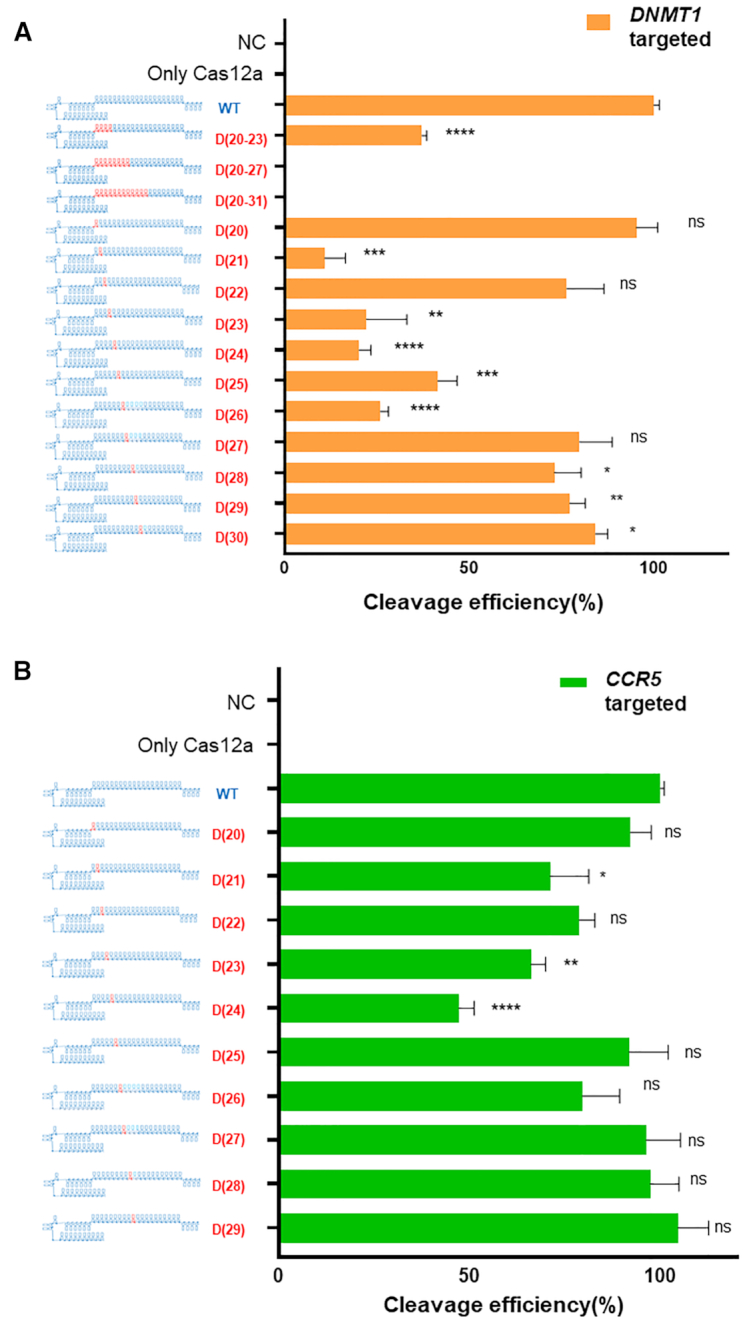
Target DNA cleavage by Cas12a using a chimeric DNA–RNA guide in which the seed region of the (cr)RNA is replaced with DNA. (**A**) Comparative analysis of the target (*DNMT1*: orange) DNA cleavage efficiency using various (cr)RNAs harboring serial multiple or single DNA substitutions in the seed region close to the PAM (TTTN). (**B**) Comparative analysis of the target (*CCR5*: green) DNA cleavage efficiency. AsCas12a (cr)RNA was serially replaced with single DNA nucleotides from the PAM. The RNA portion of the (cr)RNA is shown in blue, and the DNA portion is shown in red (‘D’ indicates a DNA and the number of substituted DNA nucleotides is indicated). All cleavage efficiency were calculated from agarose gel separated band intensity (cleaved fragment intensity (%)/total fragment intensity (%)) and normalized to wild-type (cr)RNA (Figure S1). Data are shown as means ± s.e.m. from three independent experiments. *P*-values are calculated using a two-tailed Student's t-test (ns: not significant, *P< 0.05, **P< 0.01, ***P < 0.001, ****P < 0.0001).

Next, we replaced single nucleotides in the seed region of the guide, which is located close to the PAM sequence and required for target DNA–gRNA heteroduplex formation, with DNA bases and assessed the effects on Cas12a protein activity. Target DNA cleavage experiments were performed for two genes (*DNMT1, CCR5*) (Figure 2). Target gene cleavage efficiency was reduced after DNA replacement in the seed region of (cr)RNA up to 7-nt (crRNA17-23) from the PAM for the target sequence of *DNMT1* (Figure 2A) and 5-nt distance region from the PAM (crRNA65) for *CCR5* (Figure 2B) gene respectively. In addition, according to the target sequences in *DNMT1* and *CCR5*, different patterns of cleavage efficiencies were obtained by replacing the seed-region RNA nucleotides with DNA nucleotides. Thus, the effect of DNA substitution in the (cr)RNA depends on the nucleotide sequence of the target gene. Next, we evaluated the effects of gradual DNA substitutions in groups of four nucleotides in the seed region (Figure 2A, top). As expected, this led to a greater reduction in target cleavage efficiency than single base substitution. Taken together, these findings suggested that DNA substitution in the 3′ region (up to 8 nt) better maintained the cleavage activity than DNA substitution in the seed or 5′-hairpin region of the guide. All the chimeric (cr)RNA forms evaluated (Supplementary Figure S1) were screened for Cas12a specificity in an additional on-/off-target cleavage test.

### Target DNA cleavage specificity of chimeric DNA–RNA-guided Cas12a

**Figure 3.**
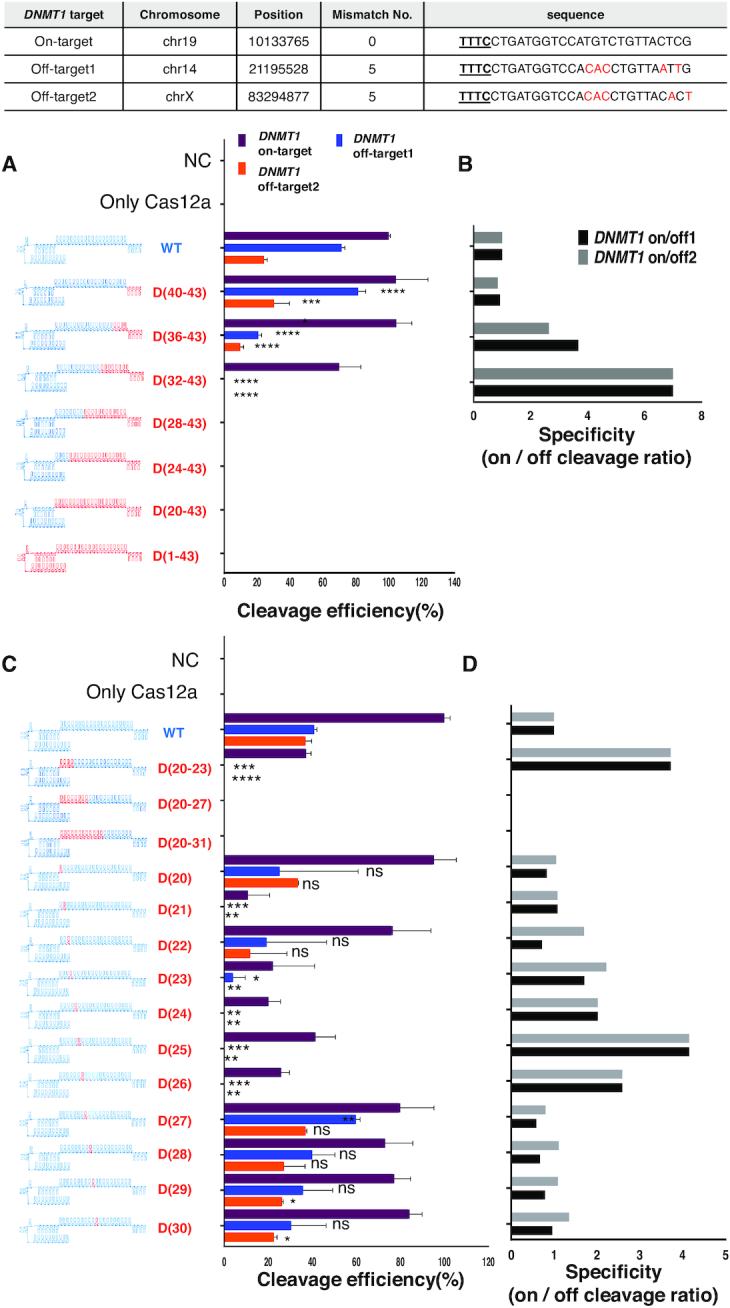
Off-target cleavage assay of Cas12a using chimeric DNA–RNA guides. (**A**) Target and off-target *DNMT1* DNA cleavage experiments to confirm the target specificity of AsCas12a using a chimeric DNA–RNA guide (serial 4-nt DNA substitutions from the 3′-end; on-target (dark brown), off-target 1 (blue), off-target 2 (orange)). The RNA portion of the Cas12a (cr)RNA is shown in blue, and the DNA portion is shown in red ('D' indicates a DNA and the position number of substituted DNA nucleotides is indicated). (**B**) Comparison of the *DNMT1* target specificity (on/off cleavage ratio (%)) of AsCas12a using chimeric DNA–RNA guides with serial 4-nt DNA substitutions from the 3′-end of the (cr)RNA (Substituted DNA nucleotides in (cr)RNA is shown in red color and RNA is shown in blue color). Target specificity was calculated from the results in (A) by dividing on-target cleavage efficiency by off-target cleavage efficiency. Target specificity is shown in black (on/off 1) and dark gray (on/off 2). (**C**) Target and off-target *DNMT1* DNA cleavage experiments to confirm the target specificity of AsCas12a using a chimeric DNA–RNA guide (DNA substitutions in the seed region of (cr)RNA; on-target (dark brown), off-target 1 (blue), off-target 2 (orange). (**D**) Comparison of the *DNMT1* target specificity of AsCas12a using chimeric DNA–RNA guides with DNA substitutions in the seed region of the (cr)RNA. Target specificity is shown in black (on/off 1) and dark gray (on/off 2). All cleavage efficiency were calculated from agarose gel separated band intensity (cleaved fragment intensity (%)/total fragment intensity (%)) and normalized to wild-type (cr)RNA. Data are shown as means ± s.e.m. from three independent experiments. *P*-values are calculated using a two-tailed Student's *t*-test (ns: not significant, *P< 0.05, **P< 0.01, ***P< 0.001, ****P< 0.0001). On- and off-target sequence information for each gene is shown in table at the top. The PAM sequence is underlined and shown in bold. Mismatch sequences to wild-type reference are shown in red.

**Figure 4.**
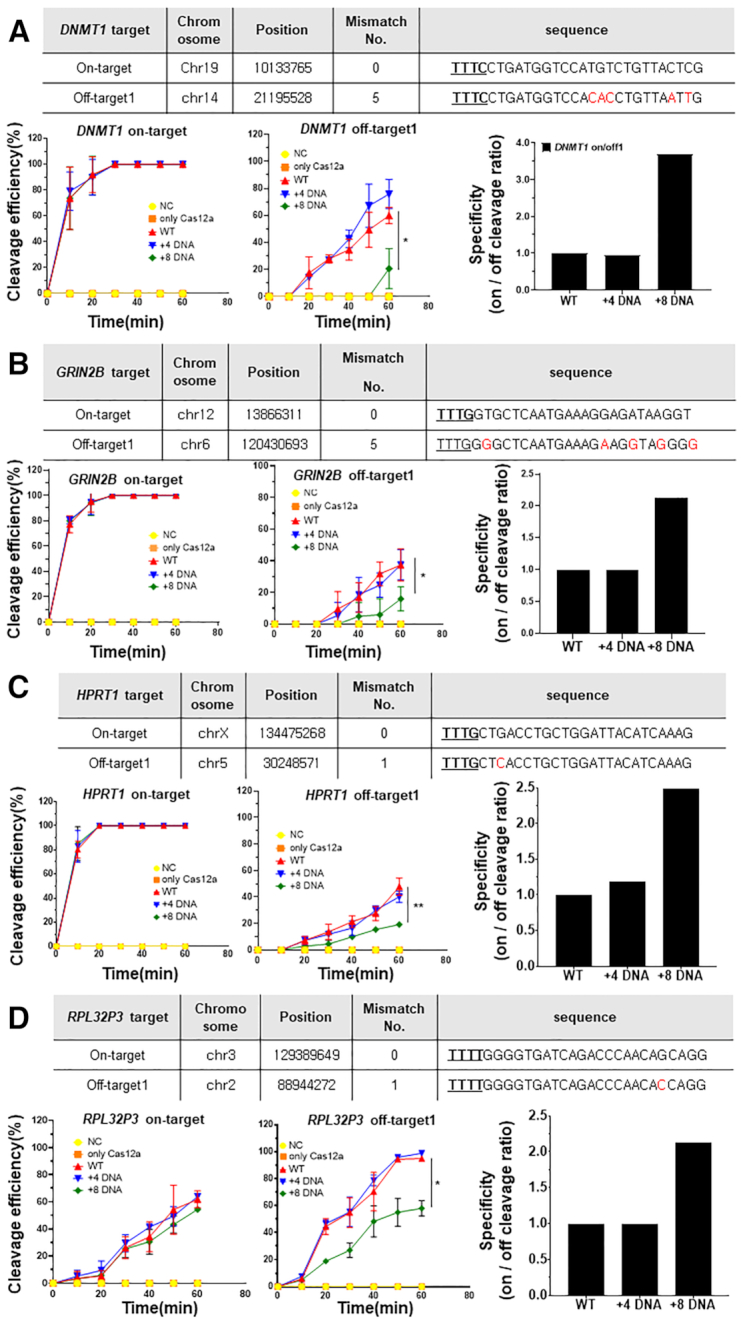
Time-course analysis of the on/off-target cleavage of Cas12a using chimeric DNA–RNA guides on various target sequences. Target and off-target DNA cleavage experiments to confirm the target specificity of AsCpf1 using a chimeric DNA–RNA guide (DNA substitutions in the 3′-end) on (**A**) *DNMT1*, (**B**) *GRIN2B*, (**C**) *HPRT1* and (**D**) *RPL32P3* genes. Table: Information about on- and off-target sequences, Left: On-target cleavage over time, Middle: Off-target cleavage over time, Right: Target specificity (on/off cleavage ratio (%)) of AsCas12a using chimeric DNA–RNA guides (serial 4-nt and 8-nt DNA substitutions in the 3′-end of the (cr)RNA). NC: negative control, only Cas12a: only protein treated, WT: Wild-type crRNA was treated with Cas12a, +4 DNA: Chimeric crRNA (sequential 4-nt DNA substitution at 3′-end of crRNA) was treated with Cas12a, +8 DNA: Chimeric crRNA (sequential 8-nt DNA substitution at 3′-end of crRNA) was treated with Cas12a. All cleavage efficiencies were calculated from agarose gel separated band intensity (cleaved fragment intensity (%)/total fragment intensity (%)) for 60 min at 10 min interval points and normalized to wild-type (cr)RNA. Data are shown as means ± s.e.m. from three independent experiments. *P*-values are calculated using a two-tailed Student's *t*-test (ns: not significant, *P< 0.05, **P< 0.01, ***P< 0.001, ****P< 0.0001). On- and off-target sequence information for each gene is shown in table at the top. The PAM sequence is underlined and shown in bold. Mismatch sequences to wild-type reference are shown in red.

To confirm the target DNA specificity after DNA substitution of the AsCas12a guide, the on- and off-target cleavage efficiencies for the *DNMT1* gene were measured (Figure 3). Off-target sequences for each target sequence with up to seven mismatches were predicted *in-silico* using Cas-OFFinder (28) (Supplementary Table S1), so that highly probable off-target cleavages were interrogated (Figure 3A, C, inset table), and the on/off cleavage ratio was calculated to compare the target specificity (Figure 3B, D). First, we measured on- and off-target cleavage efficiencies of the chimeric (cr)RNAs presented in (Figures 1, 2, Supplementary Table S1) for *DNMT1*, which revealed that two off-target sites showed different cleavage efficiency relative to the on-target sequence (Figure 3A, C). In this assay, we used low molar Cas12a concentration (molar ratio = target DNA:AsCpf1 complex = 1:2) for on-target cleavage and high molar concentration (molar ratio = target DNA:AsCpf1 complex = 1:20) for off-target cleavage not to misinterpret the cleavage efficiency due to inadequate concentration. 3′-end sequential substitution of +8 DNA (8-nt) and +12 DNA (12-nt) nucleotides in the (cr)RNA resulted in a significant decrease in cleavage at off-target sites 1 and 2, respectively (Figure 3A). In the case of 3′-end 8-nt continuous DNA substitution of the (cr)RNA, the specificity increased by 2.64–3.68-fold, without a decrease in on-target cleavage efficiency (Figure 3B). Increased target specificity was also observed for the FnCas12a effector (Supplementary Figure S4B). For *DNMT1* target and off-target amplicons, FnCas12a showed robust on-target cleavage, but almost no off-target cleavage when a 3′-end 8-nt DNA-substituted (cr)RNA was used. In the case of single-nucleotide DNA substitution in the (cr)RNA seed region, up to 11 gradual single substitutions from the PAM sequence showed a high correlation between off-target and on-target cleavage sites (Figure 3C). In particular, single-nucleotide DNA substitution in the seed region tended to significantly hamper on-target cleavage efficiency, despite the increase in target specificity due to the absence of off-target cleavage. As a result, there was a significant increase in cleavage specificity (upto 4.15-fold) for chimeric guides ((cr)RNA D20-23, D23, D24, D25, D26) that did not induce DNA off-target cleavage (Figure 3D). For the *DNMT1* target gene, consecutive DNA substitutions from the 3′-end of the guide to the eighth nucleotide maintained the on-target cleavage activity and resulted in higher target cleavage specificity than DNA substitutions in the seed region close to the PAM sequence (Figures 3B, D). A time-course cleavage analysis was performed to measure the actual full-time kinetics of on and off target DNA cleavage by the screened 3′-end DNA substituted chimeric guide (Figure 4A). As a result, when using the chimeric guide, the same cleavage kinetics were maintained at the same target sequence of the *DNMT1* gene tested in (Figure 3). It was confirmed that the cleavage kinetics were significantly reduced for off-target sequence 1 (Figure 4A, middle) and off-target sequence 2 (Supplementary Figure S5, left). The target specificity was increased to 3.68-fold (Figure 4A, right) and 7.38 fold (Supplementary Figure S5, right), each. Next, we were sought to find whether the decrease of off-target cleavage is affected by the mismatched position when using 3′-end DNA substitution of the guide (Supplementary Figure S6). According to previous studies (19,20), Cas12a has a general tolerance to the mismatched bases in the middle and PAM-distal regions of the protospacer. We performed a time-course cleavage assay by forming a randomized off-target library for the *DNMT1* target to test the overall off-target cleavage activity. As a result, +8 DNA substitution of 3′-end guide RNA reduced off-target cleavage for a randomized (12–14N) library target (Supplementary Figure S6A. On the other hand, for the 18–20N library target, the off-target cleavage was hardly reduced (Supplementary Figure S6B). Specifically, in the library targets where 12–14N and 18–20N were simultaneously randomized, the effects seen in 12–14N were disappeared by 18–20N (Supplementary Figure S6C). These data indicate that the mismatched base position in the base-pairing region of the guide RNA and target DNA strand can have a decisive effect on the off-target cleavage rate. In subsequent experiments, we used RNA–DNA chimeric guides with 3′-end substituted sequences to determine the target specificity for more gene sequences (Figures 4B–D). We performed time-course cleavage analysis for various on and off-target DNA amplicons (*GRIN2B, HPRT1* and *RPL32P3*), which has an exactly same sequence with endogenous on and off- target sites in human cells (Figure 4, Tables). When eight nucleotides were substituted at the 3′-end of the guide, on-target cleavage rate was maintained for three genes (*GRIN2B, HPRT1* and *RPL32P3*), and off-target cleavage rate was decreased relative to that of the wild-type (cr)RNA at predicted off-target sites for each target sequence (Figure 4B–D, left, middle). *In-vitro* cleavage experiments showed similar results in that the specificity (on-/off-target cleavage ratio) was substantially increased (2–2.5-fold) after 3′-end (serial 8-nt) DNA substitution of the guides for the three additional genes (Figure 4B–D, right). At other off-target sites for two target genes (*FANCF, EMX1*), mismatches in the seed region induced a weak effect of chimeric guides or inhibited off-target cleavage despite the use of a high concentration of Cas12a-guide complex (Supplementary Figure S7A, B). The use of a chimeric DNA–RNA guide rather increased on-target cleavage and resulted in high target specificity (Supplementary Figure S7C, D). These results showed that 3′-end DNA substitution of the RNA guide increases target DNA cleavage specificity without significant effect of the nucleotide sequence itself.

### 3′-End DNA-substituted chimeric DNA–RNA-guided Cas12a shows reduced activity on the endogenous locus in human cells

**Figure 5.**
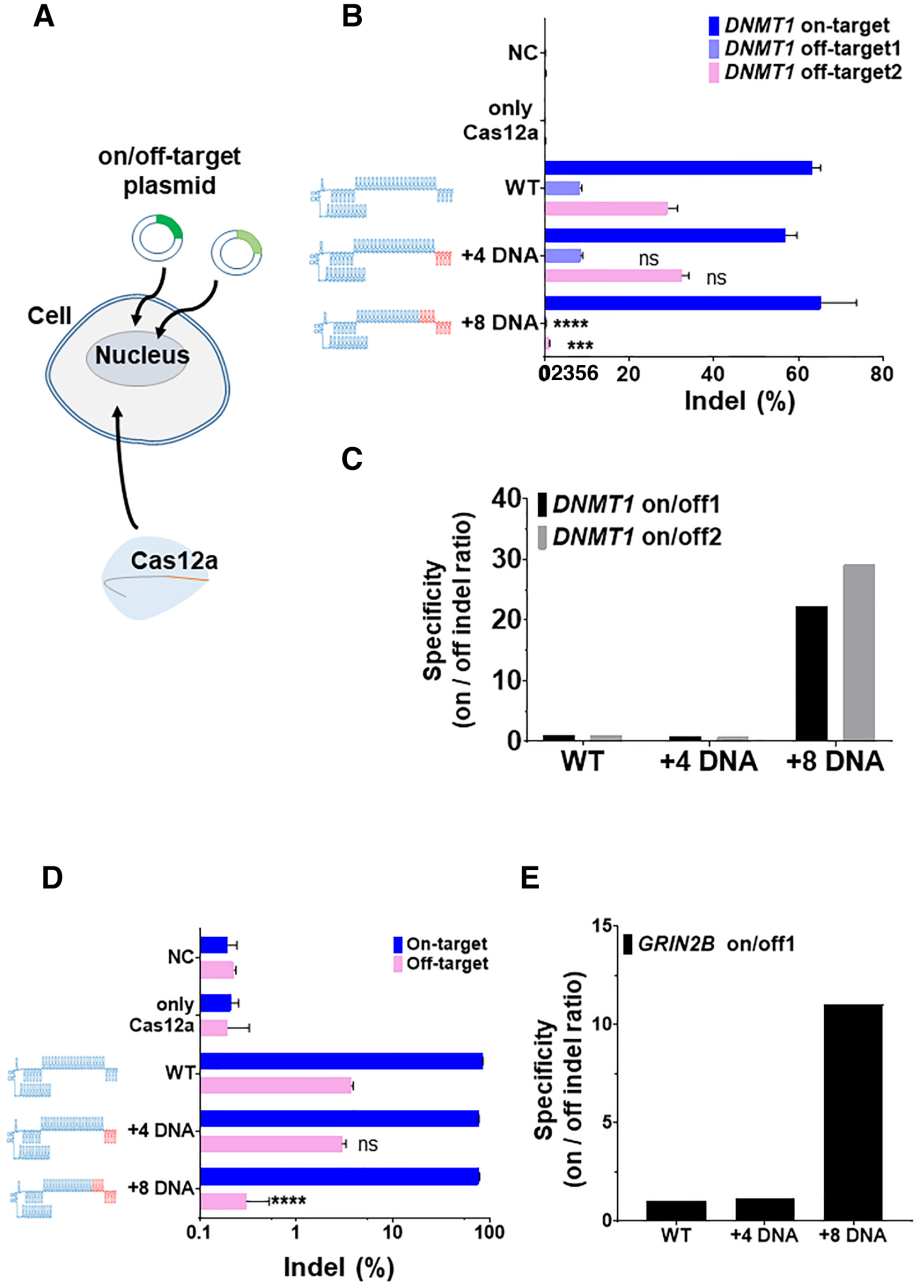
Intracellular on-/off- target sequence editing of plasmid using Cpf1 and chimeric DNA–RNA guides. (**A**) Each plasmid inserted with on-target and off-target sequences was simultaneously delivered to HEK293FT cells and targeted with Cas12a-(cr)RNA complex. (**B**) Indel ratio (%) for *DNMT1* plasmid (on-, off-target1, 2) targeted editing with chimeric (cr)RNA guided AsCas12a in HEK293FT cell. Y-axis indicates various (cr)RNAs for AsCas12a and X-axis indicates the Indel ratio (%). The RNA portion of the Cas12a (cr)RNA is shown in blue, and the substituted DNA portion is shown in red. Indel ratio (%) is calculated by targeted amplicon sequencing (NGS) from *DNMT1* site in the plasmid (indel frequency (%) = mutant DNA read number/total DNA read number). Data are shown as means ± s.e.m. from three independent experiments. *P*-values are calculated using a two-tailed Student's *t*-test (ns: not significant, *P< 0.05, **P< 0.01, ***P< 0.001, ****P< 0.0001). (**C**) Determination of target specificity (on/off cleavage ratio) calculated from (B). Target specificity is shown in black (on/off 1) and gray (on/off 2), respectively. (**D**) Indel ratio (%) for *GRIN2B* plasmid (on-, off-target1) targeted editing with chimeric (cr)RNA guided AsCas12a in HEK293FT cell. Indel frequency (%) was calculated similar to that in (B). (**E**) Determination of target specificity (on/off cleavage ratio) calculated from (D). NC: negative control, only Cas12a: only protein treated, WT: Wild-type crRNA was treated with Cas12a, +4 DNA: Chimeric crRNA (sequential 4-nt DNA substitution at 3′-end of crRNA) was treated with Cas12a, +8 DNA: Chimeric crRNA (sequential 8-nt DNA substitution at 3′-end of crRNA) was treated with Cas12a.

**Figure 6.**
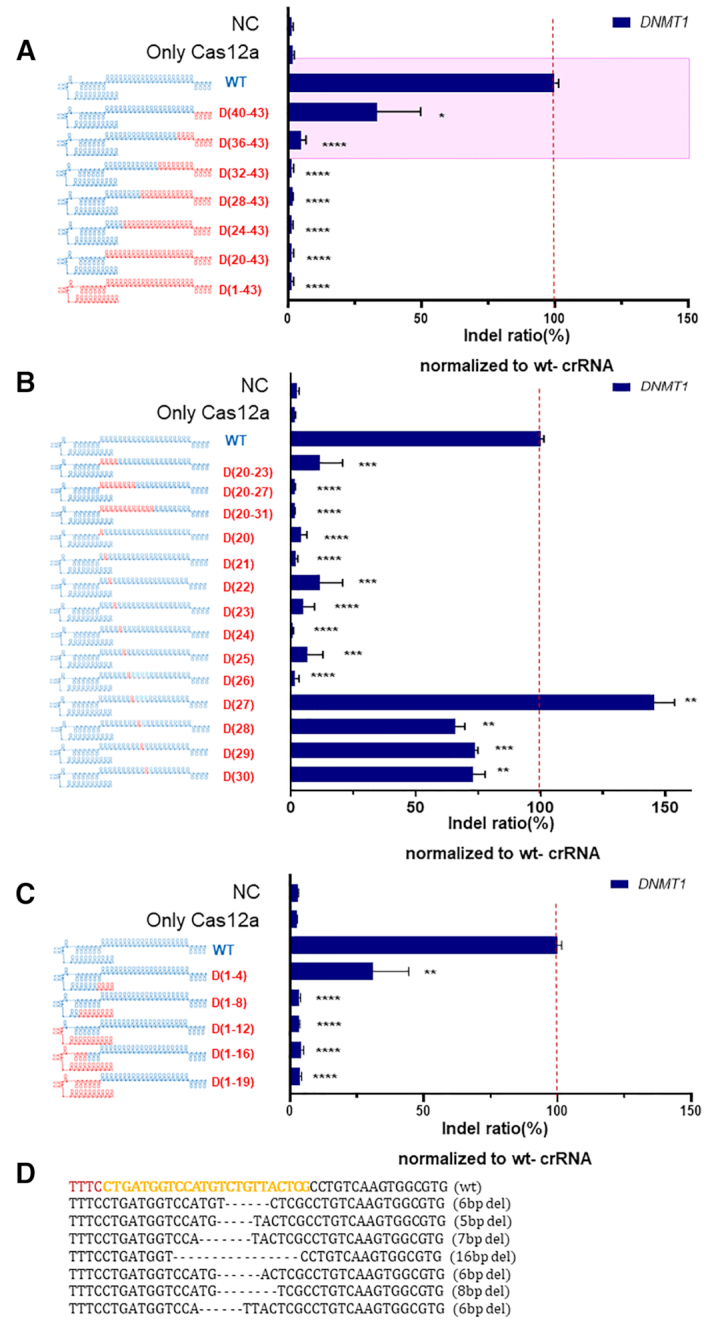
Intracellular genome editing using Cas12a and chimeric DNA–RNA guides. (**A**) Endogenous locus (*DNMT1*) editing in HEK293FT cells using chimeric DNA–RNA guides with serial 4-nt DNA substitutions from the 3′-end of the (cr)RNA. The RNA portion of the Cas12a (cr)RNA is shown in blue, and the DNA portion is shown in red ('D' indicates a DNA and the number of substituted DNA nucleotides is indicated). Cas12a genome editing with (cr)RNA with wild-type, 3′-end 4-nt DNA substitution and 3′-end 8-nt substitution is highlighted in the pink box. (**B**) Endogenous locus (*DNMT1*) editing in HEK293FT cells using chimeric DNA–RNA guides with DNA substitutions in the seed region of the (cr)RNA. (**C**) Endogenous locus (*DNMT1*) editing in HEK293FT cells using chimeric DNA–RNA guides with serial 4-nt DNA substitutions from the 5′-end of the (cr)RNA. (**D**) Representative genome editing pattern of AsCas12a using chimeric DNA–RNA guides. Sequencing data is obtained from *DNMT1* targeted amplicon sequencing (NGS). The deleted base is shown by the dashed line. PAM sequence (TTTN) and target sequence for AsCas12a is shown in brown and yellow color, respectively. All the relative indel ratio was calculated from targeted amplicon sequencing (indel frequency (%) = mutant DNA read number/total DNA read number) and normalized to wild-type (WT) (cr)RNA. Data are shown as means ± s.e.m. from three independent experiments. *P*-values are calculated using a two-tailed Student's *t*-test (ns: not significant, *P< 0.05, **P< 0.01, ***P< 0.001, ****P< 0.0001).

For the *in-vivo* application of Cas12a using various chimeric RNA–DNA guides with high target cleavage specificity, we confirmed whether target-specific genome editing could be induced at the cellular level. At first, we used the plasmid target (on/off-targets for *DNMT1, GRIN2B*), which was edited by various chimeric DNA–RNA guided Cas12a to induce the target-specific mutations (Figure 5). Indel mutations at target and *in-silico* predicted off-target sequences were investigated by delivering on and off-target plasmids into HEK293FT cells at the same time (Figure 5A). It was confirmed that the indel ratio (%) was significantly reduced at off-target 1, 2 for *DNMT1* when using 3′-end 8-nt DNA substituted chimeric (cr)RNA (Figure 5B, Supplementary Figure S8) as in the *DNMT1* off-target cleavage experiment (Figure 3A). As a result, when chimeric (cr)RNA was used intracellularly, it was confirmed that the specificity was significantly increased (22.24∼29.18 fold) (Figure 5C). In the plasmid experiment for *GRIN2B*, it was also confirmed that the chimeric DNA–RNA effect was reproduced identically as the *DNMT1* target, which shows significantly reduced off-target mutation in cells compared to on-target sequence (Figure 5D). Target specificity was increased by 11.0-fold (Figure 5E). We observed that all chimeric guide induced mutations in the target gene (*DNMT1, GRIN2B*) were mostly deletions (Supplementary Figure S8). Next, we sought to test the activity of chimeric DNA–RNA guided Cas12a on the genomic locus in human cell lines. Contrary to our expectations, 3′-end DNA substitution of the (cr)RNA led to a sharp decrease in indel formation efficiency at the target sequence (Figure 6A, boxed region, D, Supplementary Figure S9), unlike the findings in the *in-vitro* PCR amplicon (Figure 1B) or plasmid (Figure 5B) cleavage experiment. In contrast, when a seed-region DNA-substituted guide was used, indels were induced at efficiency similar to that of *in-vitro* amplicon cleavage (Figure 6B, Supplementary Figure S9). In particular, the CRISPR-Cas12a activity in cells was decreased when the DNA substitution was located in the seed region rather than in the PAM distal region (Figure 6B). In the case of DNA substitution at the 5′-end of the guide, the indel formation rate was reduced to a level similar to that of the *in-vitro* cleavage experiment (Figure 1D). This result indicated that Cas12a is highly deactivated upon alteration in the conserved 5′-end structure of (cr)RNA (Figure 6C, Supplementary Figure S9). Together, these results suggested that the difference in cleavage activity between PCR amplicons and endogenous intracellular loci might be related to various effects of the intracellular condition, such as degradation issues by DNA exonuclease (29), structural hindrances by binding of histones or other proteins (30), or the condition of intracellular chromosome topology (31), which might affect the binding of CRISPR effector proteins *in vivo* (32).

### 3′-End modification of the chimeric DNA–RNA partially recovers Cas12a activity in human cells

To determine and prevent the degradation of the chimeric guide by 3′-end DNA exonuclease (33), we performed target-specific genome editing using a chimeric guide of which the 3′ end (with 4-nt or 8-nt DNA substitution) moiety was chemically modified with phosphorothioate (PS) (Supplementary Figure S10, Supplementary Table S1). First, to test AsCas12a activity when using 3′-end-modified chimeric guides, the *DNMT1* target cleavage was assessed *in vitro* (Supplementary Figure S10A). The target DNA cleavage efficiency of the 3′-end-modified guide was similar to that of the wild-type gRNA, without reducing the on-target cleavage effect. On the other hand, upon 3′-end 8-nt DNA substitution of the guide, cleavage at off-target sites 1 and 2 were reduced or not detected, respectively (Supplementary Figure S10A). Thus, the target specificity was increased (upto 10.35-fold) as compared to that of wild-type or 3′-end +4 DNA-substituted guides, even with 3′-end modification (Supplementary Figure S10B). To evaluate Cas12a activity with chemically modified chimeric guides in mammalian cells, 3′-end modified chimeric guides and purified Cas12a proteins were delivered together into HEK293FT cells to induce indels in the target gene (Supplementary Figure S10C, D). The decrease in on-target cleavage was restored by 2.3-fold when compared to that of a non-chemically modified guide with 4-nt DNA substitution (Supplementary Figure S10C). However, in the case of 8-nt DNA substitution, the editing efficiency was not enhanced by 3′-end PS modification. These results suggested that Cas12a with 3′-end 4-nt DNA chimeric guides, for which part of the (cr)RNA is separated from the target DNA strand, can be effectively applied in cells to partially recover the genome editing activity. For 3′-end 8-nt DNA chimeric (cr)RNA, which has both base-paired and separated regions to target DNA strand, further improvement other than a 3′-end chemical modification is needed to activate the Cas12a on endogenous loci inside the cell.

### SpCas9 nickase in combination with chimeric DNA–RNA-guided Cas12a ensures highly efficient and specific genome editing in intracellular conditions

To improve the genome editing efficiency of (cr)RNA with 3′-end 8-nt DNA substitution, we tried to solve the chromosome topology issue in intracellular conditions, which possibly leads to highly compact genomic DNA and hinders the accessibility of Cas12a to the target sequence (34). Based on a previous study, we sought to relax and expose the target sequences using a dead or nickase-type CRISPR system (32). To this end, we confirmed whether the genome-editing efficiency of Cas12a using a 3′-end 4- or 8-nt DNA-substituted (cr)RNA could be improved by using it in combination with dead SpCas9 or SpCas9 nickase (Supplementary Figure S11). A single-guide RNA was designed (Supplementary Figure S11D, Supplementary Table S3) for the binding of the dead (D10A, H840A) or nickase (D10A) SpCas9 16-bp away from the *DNMT1* sequence, and purified SpCas9-sgRNA complex was transfected into cells together with Cas12-chimeric DNA–RNA complex. Interestingly, compared to only Cas12a treated cells, dead-type SpCas9-treated cells showed no significant change in target genome indel frequency (%) upon 4- or 8-nt DNA substitution of the (cr)RNA, but treatment with nickase-type SpCas9 was found to dramatically increase the indel frequency (%) in the target sequence (Supplementary Figure S11A). In particular, we observed a greater increase in indel ratio (%) of fold increase (22.1-fold) for the 8-nt DNA-substituted (cr)RNA, which contains a region that base-pairs with the target DNA strand, than for the 3′-end 4-nt DNA substituted (cr)RNA (Supplementary Figure S11B). Targeted amplicon sequencing was also performed for predicted off-target sites for the *DNMT1* target sequence similar to the in-vitro cleavage assay (Figure 3A, B), and no significant off-target indels were observed (Supplementary Figure S11C). When only dead or nickase-type SpCas9 complex was delivered, no indels were generated.

**Figure 7.**
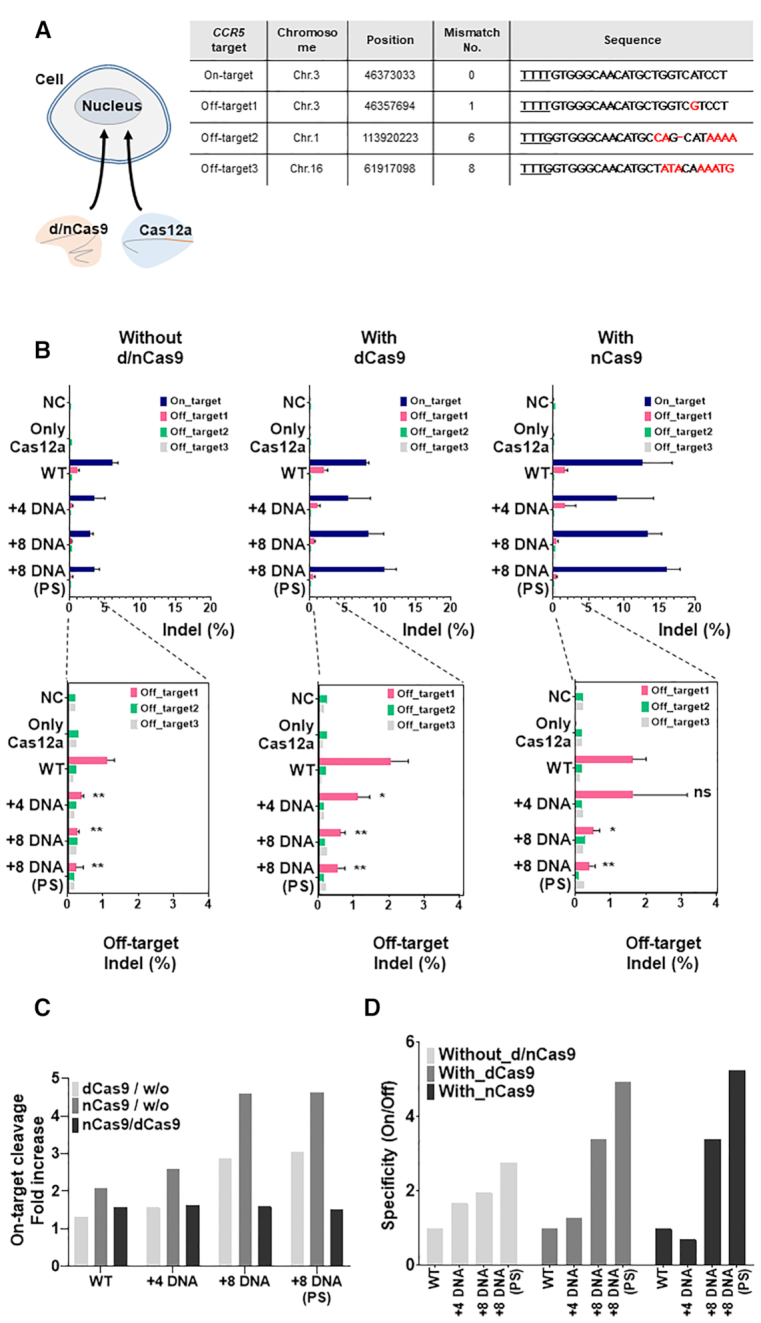
Cas12a editing efficiency and specificity are enhanced by combination of a chimeric DNA–RNA guide and dead(d) / nickase(n) form of SpCas9. (**A**) Endogenous *CCR5* gene editing in HEK293FT cell using chimeric DNA–RNA guided Cas12a and d/n-SpCas9 combination. Expression plasmids for Cas12a and d/n-SpCas9 were simultaneously transfected, then, chimeric guides were transfected sequentially. Table: Information about on- and off-target sequence for *CCR5* gene. The PAM sequence is underlined and shown in bold. Mismatch sequences to wild-type reference are shown in red. (**B**) Top: Efficiency of genome editing for on- and off-target in *CCR5* gene. X-axis indicates the indel frequency (%) and Y-axis indicates negative controls and various (cr)RNAs used for AsCas12a genome editing. Only (cr)RNA–Cas12a treated (left) and combination with dCas9 (middle) or nCas9 (right) treated samples were indicated. dCas9 and nCas9 indicate deactivated Cas9 (D10A, H840A) and nickase Cas9 (D10A), respectively. Bottom: The off-target indel frequency (%) was magnified. Indel ratio (%) is calculated by targeted amplicon sequencing from *CCR5* site in HEK293FT cells (indel frequency (%) = mutant DNA read number/total DNA read number). Data are shown as means ± s.e.m. from three independent experiments. *P*-values are calculated using a two-tailed Student's *t*-test (ns: not significant, *P< 0.05, **P< 0.01, ***P< 0.001, ****P< 0.0001). (**C**) Fold increase in the indel ratio (%) between only Cas12a treated, Cas12a and dCas9 co-treated, and Cas12a and nCas9 co-treated samples. Fold change is shown in light gray (dCas9 combination with Cas12a/only Cas12a treated), dark gray (nCas9 combination with Cas12a/only Cas12a treated) and black (nCas9 combination with Cas12a / dCas9 combination with Cas12a). (**D**) Determination of target specificity (on/off1 cleavage ratio) calculated from (B). Target specificity is shown in light gray (without d/n-Cas9 treatment), gray (Cas12a and dCas9 co-treated) and black (Cas12a and nCas9 co-treated), respectively. NC: negative control, only Cas12a: only protein treated, WT: Wild-type crRNA was treated with Cas12a, +4 DNA: Chimeric crRNA (sequential 4-nt DNA substitution at 3′-end of crRNA) was treated with Cas12a, +8 DNA: Chimeric crRNA (sequential 8-nt DNA substitution at 3′-end of crRNA) was treated with Cas12a.

**Figure 8.**
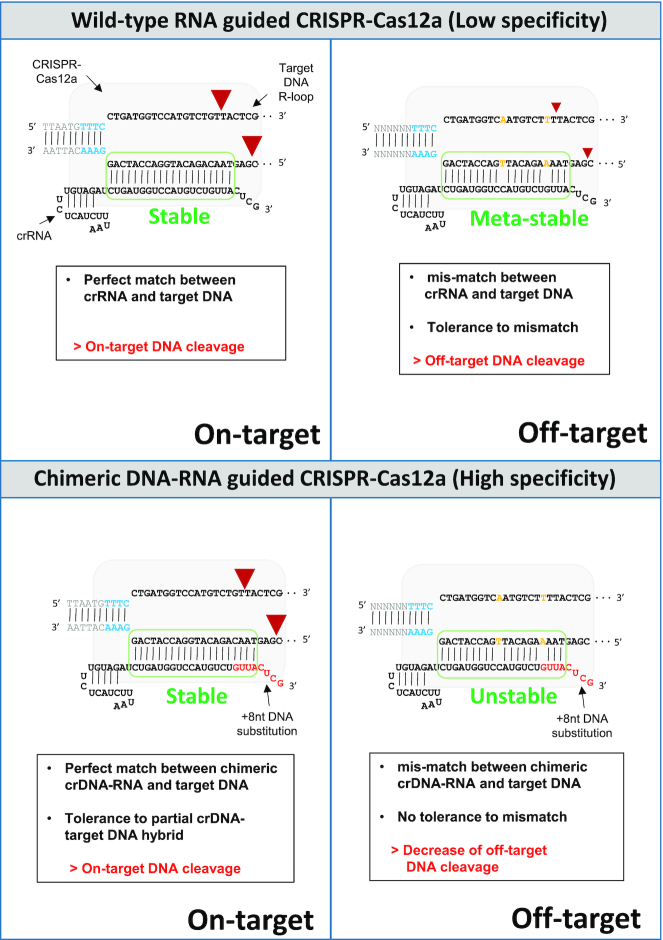
Mechanism underlying the enhanced Cas12a editing specificity by using chimeric DNA–RNA guide. In this model, when the 3′-end DNA-substituted crRNA was applied to the Cas12a system, the principle that the off-target mutation was reduced compared to wild-type crRNA was explained by the change of the hybridization energy between crRNA and target DNA strand. When wild-type crRNA is applied, crRNA-target DNA hybridization energy can endure mismatch caused by off-target binding, but when chimeric crRNA is applied, induction of off-target mutation is reduced due to destabilization of hybridization energy. The small and large red arrowheads indicate the cleavage site and degree for double-strand break by Cas12a. Red color in the (cr)RNA indicates DNA substitution. PAM sequences are shown in blue color and mismatched sequence to target sequence are shown in orange color, respectively.

To test the synergistic effect of the chimeric (cr)RNA and nickase activity on a more endogenous locus, we sequentially delivered *CCR5* targeted Cas12a and d/n SpCas9 expression vectors and the guide RNAs into the cell (Figure 7A, Supplementary Figure S12A, B). Without d/n SpCas9 treatment, weak indel mutations were generated on the endogenous *CCR5* target site by chimeric DNA–RNA guided Cas12a (Figure 7B, left). However, dead/nickase SpCas9 (D10A) and chimeric (cr)RNA showed a substantial recovery on genome editing efficiency (1.33–4.62-fold increase) of *CCR5* gene locus (Figure 7B, middle, right, C). These results suggested that Cas12a efficiently operates in a chimeric (cr)RNA-dependent manner only when a chromosome structure is locally changed and the target DNA sequence is exposed under intracellular conditions. To investigate the off-target issues generated by a combination of nickase and Cas12a effector, we also performed targeted amplicon sequencing for in-silico predicted off-target sites that correspond to the on-target sequence in *CCR5* gene (Figure 7B, bottom, Supplementary Figure S12C, D). As a result, when analyzed by targeted amplicon sequencing, it was confirmed that off-target mutation at site 1 (Figure 7A, table) for *CCR5* target was dramatically decreased in the sample treated with nickase and chimeric (cr)RNA guided Cas12a (Figure 7B, bottom). The combination of nickase and chimeric guide increased the accuracy of CCR5 gene targeting by 3.39–5.25-fold than the wild-type guide (Figure 7D). In conclusion, our data show that Cas12a works effectively with chimeric DNA–RNA guides upon locally remodeling the chromatin structure. Through the change of the hybridization energy between guide RNA and target DNA strand by DNA substitution in the (cr)RNA, the use of nickase and chimeric guide combination ensures highly-specific genome editing with Cas12a effector. On the basis of this concept, we suggest a model for the highly efficient and specific target DNA cleavage mechanism of Cas12a that operates based on a chimeric DNA–RNA guide as shown in Figure 8.

## DISCUSSION

Based on the well-defined interaction of gRNAs and specific amino acids of proteins in the well-established CRISPR–Cas12a complex with target DNA, we screened chimeric guides with high target specificity by reducing off-target cleavage without reducing on-target cleavage efficiency from various (cr)RNAs with DNA substitution at the 5′-end, 3′-end, or seed region. When various chimeric guides and Cas12a proteins were applied to off-target sequences which are similar to on-targets, the tendency shows that if the mismatch exists in the PAM-proximal seed region, as reported by previous studies on off-target detection (19,20), off-target cleavage was hardly generated. However, off-target cleavage with a mismatch in the PAM-distal region or the middle of the target sequence was frequently observed, such as those observed in *DNMT1, GRIN2B*, *HPRT1* and *RPL32P3* gene targets in this study. At this time, in the case of using continuous (+8nt) DNA substitution at the 3′-end of the guide, cleavage rate was substantially reduced in *DNMT1*, *GRIN2B, HPRT1* and *RPL32P3* off-target sites, which greatly increased the target specificity of Cas12a. On the other hand, off-target cleavage was slightly decreased or not detected, but on-target cleavage efficiency was rather increased for *FANCF* and *EMX1* gene targets; the specificity was also increased. This is thought to be a complex effect of Cas12a's structural recognition of four non-base pairing regions of (cr)RNA and change of the binding energy within 20 base-paired regions of the target sequence (Supplementary Figure S7). From a thermodynamic point of view, it can be seen that less off-target cleavage occurs when the hybridization binding energy is unstable, depending on the position of mismatch in the off-target recognized by Cas12a (Figure 8, Supplementary Figure S6). Although it was clearly shown in the structure that Cas12a interacts with the 2′-OH groups in the guide RNA only at a subset of positions, off-target cleavage is decreased at various genomic sequences that have mismatched bases and do not directly contact the DNA substitution within (cr)RNA. We think that this is probably due to the change in the energetics between the (cr)RNA and target DNA strand induced by 3′-end DNA substitution in (cr)RNA. Among off-target sequences similar to on-target sequences, mismatched bases can affect the entire hybridization energy formed by the DNA target strand and (cr)RNA base-pairs. Therefore, as shown in the suggested model (Figure 8), when using a chimeric RNA–DNA guide, it is possible to reduce off-target cleavage by inducing instability in off-target DNA binding. Previous studies of DNA-modified guides for Cas9 have also shown that the direct contribution of the molecular contacts between Cas9 and the 2′-OH groups in the guide RNA toward DNA binding and cleavage is very small (21,35). These examples explain the phenomenon that the mismatched base in the off-target and the 3′-end region substituted by DNA in the crRNA actually does not affect the DNA cleavage even if it is not directly contacted by the Cas12a residue. Unlike the 3′-end, the DNA substitution on the 5′-hairpin seems to have a significant effect on the Cas12a activity by inducing structural changes in the hairpin region. In addition to the 5′-end hairpin structure, DNA substitution in the seed region inside the target sequence of the RNA guide significantly reduces on-target cleavage activity which indicates that Cas12a protein requires 2′-OH recognition in the seed region to stably interact with the target DNA - (cr)RNA duplex structure.

When the genome editing was induced in the cell using highly accurate chimeric (cr)RNA with 3′-end DNA substitution, we observed a decrease in endonuclease activity which is different from the result of naked PCR amplicon or plasmid cleavage. The recovery of Cas12a activity suggests that low activity with chimeric DNA–RNA guided Cas12a is possibly due to the effects of target site recognition on the intracellular genomic DNA. In this study, the combinatorial use of SpCas9 nickase and Cas12a for genome editing solved the low on-target editing efficiency issue on the intracellular condition, thus improved target-specific genome editing efficiency. In particular, we can decrease the off-target site editing efficiency of Cas12a by using a chimeric DNA–RNA guide as in our model (Figure 8). The target DNA specificity is greatly improved because chimeric guides that reduce the hybridization energy and increase the sensitivity to mismatches. By using SpCas9 nickase and chimeric (cr)RNA guided Cas12a in combination, we have developed a highly target-specific genome editing technique that possibly reduces unwanted off-target cleavages. Overall, future structural and biochemical studies on chimeric DNA–RNA recognition of the CRISPR-Cas12a protein are required to improve the target DNA cleavage efficiency by protein engineering. In particular, if the target-specific genome editing efficiency can be improved by changing the amino acid residue(s) in Cas12a required for full DNA recognition, non-specific cleavage can be dramatically reduced *in vivo*, and thus, the safety of target-specific genome editing would increase.

In this study, we suggested that the target DNA sequence specificity of Cas12a was improved by partial substitution of the (cr)RNA with DNA. We further increased the possibility of target-specific genome editing by enhancing the intracellular operating efficiency of the chimeric guide based Cas12a using the combination of nickase Cas9. The results of this study will aid in the development of chimeric DNA–RNA-guided target-specific Cas12a genome editing that can be applied in various living organisms, including microorganisms, plants, animals, and ultimately, human beings. Obviously, safety issues remain to be addressed in clinical trials.

## DATA AVAILABILITY

Targeted deep sequencing data are available at NCBI Sequence Read Archive (SRA) under accession number SRP247270. CRISPR RGEN Tools is an open-source collaborative initiative available in the repository (http://www.rgenome.net/).

## Supplementary Material

gkaa605_Supplemental_File
