# Supplementary material for: Enhancement of target specificity of CRISPR–Cas12a by using a chimeric DNA–RNA guide
Source: Nucleic Acids Res. 2020 Jul 20;48(15):8601–16. doi: 10.1093/nar/gkaa605 (PMC7470973; doi:10.1093/nar/gkaa605)
Supplement: gkaa605_Supplemental_File [file gkaa605_supplemental_file.docx]

**Supplementary Data**

**Enhancement of Target Specificity of CRISPR-Cas12a by Using a Chimeric DNA-RNA Guide**

Hanseop Kim, Wi-jae Lee, Yeounsun Oh, Seung-Hun Kang, Junho K. Hur, Hyomin Lee, WooJeung Song, Kyung-Seob Lim, Young-Ho Park, Bong-Seok Song, Yeung Bae Jin, Bong-Hyun Jun, Cheulhee Jung, Dong-Seok Lee, Sun-Uk Kim, and Seung Hwan Lee

**Supplementary Tables**

**Table S1. Sequence information for chimeric DNA-RNA guides used in this study.**

| Target gene  (guide no.) | CRISPR-Cas12a (AsCpf1)  target sequence (5′-3′) | crRNA sequence for AsCpf1 (5′-3′) |
| --- | --- | --- |
| hDNMT1 **crRNA1** | **TTTC**CTGATGGTCCATGTCTGTTACTCG | **5’AAUUUCUACUCUUGUAGAUCUGAUGGUCCAUGUCUGUUACUCG** 3’ |
| hDNMT1 **crRNA2** | **TTTC**CTGATGGTCCATGTCTGTTACTCG | **5’AAUUUCUACUCUUGUAGAUCUGAUGGUCCAUGUCUGUUACTCG 3’** |
| hDNMT1 **crRNA2-2** | **TTTC**CTGATGGTCCATGTCTGTTACTCG | **5’AATTUCUACUCUUGUAGAUCUGAUGGUCCAUGUCUGUUACTCG 3’** |
| hDNMT1 **crRNA2-3** | **TTTC**CTGATGGTCCATGTCTGTTACTCG | **5’AATTTCTACUCUUGUAGAUCUGAUGGUCCAUGUCUGUUACTCG 3’** |
| hDNMT1 **crRNA3** | **TTTC**CTGATGGTCCATGTCTGTTACTCG | **5’AAUUUCUACUCUUGUAGAUCUGAUGGUCCAUGUCUGTTACTCG 3’** |
| hDNMT1 **crRNA3-2** | **TTTC**CTGATGGTCCATGTCTGTTACTCG | **5’AAUUUCUACUCUUGUAGAUCUGAUGGUCCAUGUCTGTTACTCG 3’** |
| hDNMT1 **crRNA3-3** | **TTTC**CTGATGGTCCATGTCTGTTACTCG | **5’AAUUUCUACUCUUGUAGAUCUGAUGGUCCAUGUCTGTTACTCG 3’** |
| hDNMT1 **crRNA3-4** | **TTTC**CTGATGGTCCATGTCTGTTACTCG | **5’AAUUUCUACUCUUGUAGAUCUGAUGGUCCAUGTCTGTTACTCG 3’** |
| hDNMT1 **crRNA3-5** | **TTTC**CTGATGGTCCATGTCTGTTACTCG | **5’AATTUCUACUCUUGUAGAUCUGAUGGUCCAUGUCUGTTACTCG 3’** |
| hDNMT1 **crRNA3-6** | **TTTC**CTGATGGTCCATGTCTGTTACTCG | **5’AATTTCTACUCUUGUAGAUCUGAUGGUCCAUGUCUGTTACTCG 3’** |
| hDNMT1 **crRNA4** | **TTTC**CTGATGGTCCATGTCTGTTACTCG | **5’AAUUUCUACUCUUGUAGAUCUGAUGGUCCAUGTCTGTTACTCG 3’** |
| hDNMT1 **crRNA5** | **TTTC**CTGATGGTCCATGTCTGTTACTCG | **5’AAUUUCUACUCUUGUAGAUCUGAUGGUCCATGTCTGTTACTCG 3’** |
| hDNMT1 **crRNA6** | **TTTC**CTGATGGTCCATGTCTGTTACTCG | **5’AAUUUCUACUCUUGUAGAUCUGATGGTCCATGTCTGTTACTCG 3’** |
| hDNMT1 **crRNA7** | **TTTC**CTGATGGTCCATGTCTGTTACTCG | **5’AAUUUCUACUCUUGUAGAUCTGATGGTCCATGTCTGTTACTCG 3’** |
| hDNMT1 **crRNA8** | **TTTC**CTGATGGTCCATGTCTGTTACTCG | **5’AATTTCTACTCTTGTAGATCTGATGGTCCATGTCTGTTACTCG 3’** |
| hDNMT1 **crRNA9** | **TTTC**CTGATGGTCCATGTCTGTTACTCG | **5’AATTUCUACUCUUGUAGAUCUGAUGGUCCAUGUCUGUUACUCG 3’** |
| hDNMT1 **crRNA10** | **TTTC**CTGATGGTCCATGTCTGTTACTCG | **5’AATTTCTACUCUUGUAGAUCUGAUGGUCCAUGUCUGUUACUCG 3’** |
| hDNMT1 **crRNA11** | **TTTC**CTGATGGTCCATGTCTGTTACTCG | **5’AATTTCTACTCTUGUAGAUCUGAUGGUCCAUGUCUGUUACUCG 3’** |
| hDNMT1 **crRNA12** | **TTTC**CTGATGGTCCATGTCTGTTACTCG | **5’AATTTCTACTCTTGTAGAUCUGAUGGUCCAUGUCUGUUACUCG 3’** |
| hDNMT1 **crRNA13** | **TTTC**CTGATGGTCCATGTCTGTTACTCG | **5’AATTTCTACTCTTGTAGATCUGAUGGUCCAUGUCUGUUACUCG 3’** |
| hDNMT1 **crRNA14** | **TTTC**CTGATGGTCCATGTCTGTTACTCG | **5’AAUUUCUACUCUUGUAGAUCTGAUGGUCCAUGUCUGUUACUCG 3’** |
| hDNMT1 **crRNA15** | **TTTC**CTGATGGTCCATGTCTGTTACTCG | **5’AAUUUCUACUCUUGUAGAUCTGATGGTCCAUGUCUGUUACUCG 3’** |
| hDNMT1 **crRNA16** | **TTTC**CTGATGGTCCATGTCTGTTACTCG | **5’AAUUUCUACUCUUGUAGAUCTGATGGTCCATGUCUGUUACUCG 3’** |
| hDNMT1 **crRNA17** | **TTTC**CTGATGGTCCATGTCTGTTACTCG | **5’AAUUUCUACUCUUGUAGAUCUGAUGGUCCAUGUCUGUUACUCG 3’** |
| hDNMT1 **crRNA18** | **TTTC**CTGATGGTCCATGTCTGTTACTCG | **5’AAUUUCUACUCUUGUAGAUCTGAUGGUCCAUGUCUGUUACUCG 3’** |
| hDNMT1 **crRNA19** | **TTTC**CTGATGGTCCATGTCTGTTACTCG | **5’AAUUUCUACUCUUGUAGAUCUGAUGGUCCAUGUCUGUUACUCG 3’** |
| hDNMT1 **crRNA20** | **TTTC**CTGATGGTCCATGTCTGTTACTCG | **5’AAUUUCUACUCUUGUAGAUCUGAUGGUCCAUGUCUGUUACUCG 3’** |
| hDNMT1 **crRNA21** | **TTTC**CTGATGGTCCATGTCTGTTACTCG | **5’AAUUUCUACUCUUGUAGAUCUGATGGUCCAUGUCUGUUACUCG 3’** |
| hDNMT1 **crRNA22** | **TTTC**CTGATGGTCCATGTCTGTTACTCG | **5’AAUUUCUACUCUUGUAGAUCUGAUGGUCCAUGUCUGUUACUCG 3’** |
| hDNMT1 **crRNA23** | **TTTC**CTGATGGTCCATGTCTGTTACTCG | **5’AAUUUCUACUCUUGUAGAUCUGAUGGUCCAUGUCUGUUACUCG 3’** |
| hDNMT1 **crRNA24** | **TTTC**CTGATGGTCCATGTCTGTTACTCG | **5’AAUUUCUACUCUUGUAGAUCUGAUGGTCCAUGUCUGUUACUCG 3’** |
| hDNMT1 **crRNA25** | **TTTC**CTGATGGTCCATGTCTGTTACTCG | **5’AAUUUCUACUCUUGUAGAUCUGAUGGUCCAUGUCUGUUACUCG 3’** |
| hDNMT1 **crRNA26** | **TTTC**CTGATGGTCCATGTCTGTTACTCG | **5’AAUUUCUACUCUUGUAGAUCUGAUGGUCCAUGUCUGUUACUCG 3’** |
| hDNMT1 **crRNA27** | **TTTC**CTGATGGTCCATGTCTGTTACTCG | **5’AAUUUCUACUCUUGUAGAUCUGAUGGUCCAUGUCUGUUACUCG 3’** |
| hCCR5  **crRNA51** | **TTTG**TGCACAGGGTGGAACAAGATGGAT | **5’AAUUUCUACUCUUGUAGAUUGCACAGGGUGGAACAAGAUGGAU 3’** |
| hCCR5  **crRNA52** | **TTTG**TGCACAGGGTGGAACAAGATGGAT | **5’AAUUUCUACUCUUGUAGAUUGCACAGGGUGGAACAAGAUGGAT 3’** |
| hCCR5  **crRNA53** | **TTTG**TGCACAGGGTGGAACAAGATGGAT | **5’AAUUUCUACUCUUGUAGAUUGCACAGGGUGGAACAAGATGGAT 3’** |
| hCCR5  **crRNA54** | **TTTG**TGCACAGGGTGGAACAAGATGGAT | **5’AAUUUCUACUCUUGUAGAUUGCACAGGGUGGAACAAGATGGAT 3’** |
| hCCR5  **crRNA55** | **TTTG**TGCACAGGGTGGAACAAGATGGAT | **5’AAUUUCUACUCUUGUAGAUUGCACAGGGTGGAACAAGATGGAT 3’** |
| hCCR5  **crRNA56** | **TTTG**TGCACAGGGTGGAACAAGATGGAT | **5’AAUUUCUACUCUUGUAGAUUGCACAGGGTGGAACAAGATGGAT 3’** |
| hCCR5  **crRNA57** | **TTTG**TGCACAGGGTGGAACAAGATGGAT | **5’AAUUUCUACUCUUGUAGAUTGCACAGGGTGGAACAAGATGGAT 3’** |
| hCCR5  **crRNA58** | **TTTG**TGCACAGGGTGGAACAAGATGGAT | **5’AATTTCTACTCTTGTAGATTGCACAGGGTGGAACAAGATGGAT 3’** |
| hCCR5  **crRNA61** | **TTTG**TGCACAGGGTGGAACAAGATGGAT | **5’AAUUUCUACUCUUGUAGAUTGCACAGGGUGGAACAAGAUGGAU 3’** |
| hCCR5  **crRNA62** | **TTTG**TGCACAGGGTGGAACAAGATGGAT | **5’AAUUUCUACUCUUGUAGAUUGCACAGGGUGGAACAAGAUGGAU 3’** |
| hCCR5  **crRNA63** | **TTTG**TGCACAGGGTGGAACAAGATGGAT | **5’AAUUUCUACUCUUGUAGAUUGCACAGGGUGGAACAAGAUGGAU 3’** |
| hCCR5  **crRNA64** | **TTTG**TGCACAGGGTGGAACAAGATGGAT | **5’AAUUUCUACUCUUGUAGAUUGCACAGGGUGGAACAAGAUGGAU 3’** |
| hCCR5  **crRNA65** | **TTTG**TGCACAGGGTGGAACAAGATGGAT | **5’AAUUUCUACUCUUGUAGAUUGCACAGGGUGGAACAAGAUGGAU 3’** |
| hCCR5  **crRNA66** | **TTTG**TGCACAGGGTGGAACAAGATGGAT | **5’AAUUUCUACUCUUGUAGAUUGCACAGGGUGGAACAAGAUGGAU 3’** |
| hCCR5  **crRNA67** | **TTTG**TGCACAGGGTGGAACAAGATGGAT | **5’AAUUUCUACUCUUGUAGAUUGCACAGGGUGGAACAAGAUGGAU 3’** |
| hCCR5  **crRNA68** | **TTTG**TGCACAGGGTGGAACAAGATGGAT | **5’AAUUUCUACUCUUGUAGAUUGCACAGGGUGGAACAAGAUGGAU 3’** |
| hCCR5  **crRNA69** | **TTTG**TGCACAGGGTGGAACAAGATGGAT | **5’AAUUUCUACUCUUGUAGAUUGCACAGGGUGGAACAAGAUGGAU 3’** |
| hCCR5  **crRNA70** | **TTTG**TGCACAGGGTGGAACAAGATGGAT | **5’AAUUUCUACUCUUGUAGAUUGCACAGGGTGGAACAAGAUGGAU 3’** |
| hFANCF  **crRNA81** | **TTTG**GTCGGCATGGCCCCATTCGCACGG | **5’AAUUUCUACUCUUGUAGAUGUCGGCAUGGCCCCAUUCGCACGG 3’** |
| hFANCF  **crRNA82** | **TTTG**GTCGGCATGGCCCCATTCGCACGG | **5’AAUUUCUACUCUUGUAGAUGUCGGCAUGGCCCCAUUCGCACGG 3’** |
| hFANCF  **crRNA82-2** | **TTTG**GTCGGCATGGCCCCATTCGCACGG | **5’AAUUUCUACUCUUGUAGAUGUCGGCAUGGCCCCAUTCGCACGG 3’** |
| hGRIN2B  **crRNA83** | **TTTG**GTGCTCAATGAAAGGAGATAAGGT | **5’AAUUUCUACUCUUGUAGAUGUGCUCAAUGAAAGGAGAUAAGGU 3’** |
| hGRIN2B  **crRNA84** | **TTTG**GTGCTCAATGAAAGGAGATAAGGT | **5’AAUUUCUACUCUUGUAGAUGUGCUCAAUGAAAGGAGAUAAGGT 3’** |
| hGRIN2B  **crRNA84-2** | **TTTG**GTGCTCAATGAAAGGAGATAAGGT | **5’AAUUUCUACUCUUGUAGAUGUGCUCAAUGAAAGGAGATAAGGT 3’** |
| hEMX1  **crRNA85** | **TTTG**TCCTCCGGTTCTGGAACCACACCT | **5’AAUUUCUACUCUUGUAGAU**U**CCUCCGGUUCUGGAACCACACCU 3’** |
| hEMX1  **crRNA86** | **TTTG**TCCTCCGGTTCTGGAACCACACCT | **5’AAUUUCUACUCUUGUAGAU**U**CCUCCGGUUCUGGAACCACACCT 3’** |
| hEMX1  **crRNA86-2** | **TTTG**TCCTCCGGTTCTGGAACCACACCT | **5’AAUUUCUACUCUUGUAGAU**U**CCUCCGGUUCUGGAACCACACCT 3’** |
| hDNMT1  **crRNA2-PS** | **TTTC**CTGATGGTCCATGTCTGTTACTCG | **5’AAUUUCUACUCUUGUAGAUCUGAUGGUCCAUGUCUGUUACTCG (PS)3’** |
| hDNMT1 **crRNA3-PS** | **TTTC**CTGATGGTCCATGTCTGTTACTCG | **5’AAUUUCUACUCUUGUAGAUCUGAUGGUCCAUGUCUGTTACTCG (PS)3’** |
| hCCR5-2 **crRNA87-WT** | **TTTT**GTGGGCAACATGCTGGTCATCCTC | **5’AAUUUCUACUCUUGUAGAUGUGGGCAACAUGCUGGUCAUCCUC 3'** |
| hCCR5-2 **crRNA88_**  **+4DNA** | **TTTT**GTGGGCAACATGCTGGTCATCCTC | **5’AAUUUCUACUCUUGUAGAUGUGGGCAACAUGCUGGUCAUCCTC 3'** |
| hCCR5-2 **crRNA89_**  **+8DNA** | **TTTT**GTGGGCAACATGCTGGTCATCCTC | **5’AAUUUCUACUCUUGUAGAUGUGGGCAACAUGCUGGTCATCCTC 3'** |
| hCCR5-2 **crRNA90_**  **+8DNA(PS)** | **TTTT**GTGGGCAACATGCTGGTCATCCTC | **5’AAUUUCUACUCUUGUAGAUGUGGGCAACAUGCUGGTCATCCTC(PS) 3'** |
| hHPRT1 **crRNA91-WT** | **TTTG**CTGACCTGCTGGATTACATCAAAG | **5’AAUUUCUACUCUUGUAGAUCUGACCUGCUGGAUUACAUCAAAG 3'** |
| hHPRT1 **crRNA92_**  **+4DNA** | **TTTG**CTGACCTGCTGGATTACATCAAAG | **5’AAUUUCUACUCUUGUAGAUCUGACCUGCUGGAUUACAUCAAAG 3'** |
| hHPRT1 **crRNA93_**  **+8DNA** | **TTTG**CTGACCTGCTGGATTACATCAAAG | **5’AAUUUCUACUCUUGUAGAUCUGACCUGCUGGAUUACATCAAAG 3'** |
| hHPRT1 **crRNA94_**  **+8DNA(PS)** | **TTTG**CTGACCTGCTGGATTACATCAAAG | **5’AAUUUCUACUCUUGUAGAUCUGACCUGCUGGAUUACATCAAAG(PS) 3'** |
| hRPL32P3 **crRNA95-WT** | **TTTT**GGGGTGATCAGACCCAACAGCAGG | **5’AAUUUCUACUCUUGUAGAUGGGGUGAUCAGACCCAACAGCAGG 3'** |
| hRPL32P3 **crRNA96_**  **+4DNA** | **TTTT**GGGGTGATCAGACCCAACAGCAGG | **5’AAUUUCUACUCUUGUAGAUGGGGUGAUCAGACCCAACAGCAGG 3'** |
| hRPL32P3 **crRNA97_**  **+8DNA** | **TTTT**GGGGTGATCAGACCCAACAGCAGG | **5’AAUUUCUACUCUUGUAGAUGGGGUGAUCAGACCCAACAGCAGG 3'** |
| hRPL32P3 **crRNA98_**  **+8DNA(PS)** | **TTTT**GGGGTGATCAGACCCAACAGCAGG | **5’AAUUUCUACUCUUGUAGAUGGGGUGAUCAGACCCAACAGCAGG(PS) 3'** |

†PAM sequences (TTTN) for AsCpf1 in the target DNA are shown in blue and substituted DNA sequences in (cr)RNA targets are shown in red, respectively. PS indicates a 3’-end modification of the (cr)RNA with phosphorothioate.

**Table S2. Sequence information for DNA primers used in this study.**

| Target gene  (primer direction) | DNA sequence (5′ to 3′) |
| --- | --- |
| hDNMT1 on-target (F1) | GTTGCACGTGTCAAGTGCTTA |
| hDNMT1 on-target R1 | TTAAAATCCAGAATGCACAAAGTACT |
| hDNMT1 on-target F2 | GTGAATTTGGCTCAGCAGGCA |
| hDNMT1 on-target R2 | AAGCGAACCTCACACAACAGC |
| hDNMT1 off-target1 F1 | TTGACGGCAGTATTACAGGTAG |
| hDNMT1 off-target1 R1 | AAGGTCAAATGCCGTTTAACCA |
| hDNMT1 off-target1 F2 | GTAGTCAGGCATGAGTGGCA |
| hDNMT1 off-target1 R2 | TGCCTCTTTCCCAGGATTCT |
| hDNMT1 off-target2 F1 | TCTCAGGCAAGTCACAACTCT |
| hDNMT1 off-target2 R1 | TAGGCACATGAAGGTCAAATGC |
| hDNMT1 off-target2 F2 | GTTACAGGTAGTTAAGCAGGCA |
| hDNMT1 off-target2 R2 | AAATGCCATATTTAACCGTGATCCT |
| hDNMT1 off-target3 F1 | TCATGCCTTTCTGGGTCTCAT |
| hDNMT1 off-target3 R1 | CACCTGACCTCTGTCACTTTA |
| hDNMT1 off-target3 F2 | CTGTTCAGGGAATGGAAAGTGA |
| hDNMT1 off-target3 R2 | CTACATCTACGCTCTCCCCA |
| hCCR5-1 on-target F1 | AAGGCTGAGCTGCACCATGC |
| hCCR5-1 on-target R1 | AGGATGATGAAGAAGATTCCA |
| hCCR5-1 on-target F2 | CATTCACTCCATGGTGCTATA |
| hCCR5-1 on-target R2 | ATAGAGCCCTGTCAAGAGTTGA |
| hCCR5-1 off-target1 F1 | AGAAATAGGAGTCTTCATGCCC |
| hCCR5-1 off-target1 R1 | TGGGGTCAATGAGAGGAGTATT |
| hCCR5-1 off-target1 F2 | TAGCAAGGAACCTCAAAGTGC |
| hCCR5-1 off-target1 R2 | CTGCTTCCAATACAATCCACAC |
| hCCR5-1 off-target2 F1 | TAGTGCTGAAAGCTCAGAGAG |
| hCCR5-1 off-target2 R1 | TTCGAGAGCCACACATGAAG |
| hCCR5-1 off-target2 F2 | AGAGAGGGGGTCATAGACTTT |
| hCCR5-1 off-target2 R2 | ACCTTCCAGCCGGTATATTAAT |
| hFANCF on-target F1 | TGAAAGCGGAAGTAGGGCCT |
| hFANCF on-target R1 | CTCCGCCTGGGTCTTCATCA |
| hFANCF on-target F2 | GGAAGTAGGGCCTTCGCGCA |
| hFANCF on-target R2 | CCTCCTGGAGATTTGGGTTC |
| hFANCF off-target1 F1 | CCCCGCTGAGATCATACTAT |
| hFANCF off-target1 R1 | GAATGGAAGTTTGAGGGTAG |
| hFANCF off-target1 F2 | GGTCCAGACACTAACAATTC |
| hFANCF off-target1 R2 | GATTGCTCACAAATATGGGA |
| hFANCF off-target2 F1 | TGGGGTAGGTCTTCAGGAAA |
| hFANCF off-target2 R1 | CGTTCTAAATTCTCTACAGTCAAC |
| hFANCF off-target2 F2 | TCGTGCTAAGTTACCGAGTT |
| hFANCF off-target2 R2 | GCAATAGTTTTGAGCCAGTGA |
| hGRIN2B on-target F1 | ACCTCTGCTGAGCACGTTTT |
| hGRIN2B on-target R1 | GACAGCAATGCCAATGCTGG |
| hGRIN2B on-target F2 | CTCACTTTGTCTGGCCTTGC |
| hGRIN2B on-target R2 | CTTCTGAGAACGAGCTCTGC |
| hGRIN2B off-target1 F1 | TGACTGCAATTTTGGGTTCCATC |
| hGRIN2B off-target1 R1 | GCCACTGCCATTTATTATGTAAC |
| hGRIN2B off-target1 F2 | GTCTAATTACACTTGCCATACCT |
| hGRIN2B off-target1 R2 | GAAATCCAGCTGTGACTAAATATG |
| hGRIN2B off-target2 F1 | GGAGCACAATGAGTGTTTTT |
| hGRIN2B off-target2 R1 | GTCTTTTCCTGGTCACATGG |
| hGRIN2B off-target2 F2 | GCCAGAAATGTTATTCCATAGTAG |
| hGRIN2B off-target2 R2 | CAGTGTTCTTACATATCAGACAC |
| hEMX1 on-target F1 | ACTACTCACATCCACTCTGTGAAG |
| hEMX1 on-target R1 | GAGTGGCCAGAGTCCAGCTT |
| hEMX1 on-target F2 | TAGAGGAGCTAGGATGCACAGCA |
| hEMX1 on-target R2 | GCAGCAAGCAGCACTCTGCC |
| hEMX1 off-target1 F1 | CAGAGCCTGGAGAATTGATAG |
| hEMX1 off-target1 R1 | CTGGGGAGCAGAATAAATTATTG |
| hEMX1 off-target1 F2 | GAATAGACCTGGGATGTGCAG |
| hEMX1 off-target1 R2 | GGAACCTGAAGAATGGGATTG |
| hEMX1 off-target2 F1 | AGATTGAGATCATCCACCCT |
| hEMX1 off-target2 R1 | AAAACAGAGAGGTTGAGTCTC |
| hEMX1 off-target2 F2 | TGTCCACCATGTCCCAGAAG |
| hEMX1 off-target2 R2 | ACAAAGAAGGGCATCTCCAG |
| hCCR5-2 on-target F1 | TGAGATGGTGCTTTCATGAAT |
| hCCR5-2 on-target_R1 | GAAAATGAGAGCTGCAGGTG |
| hCCR5-2 on-target_F2 | AAACTTCATTGCTTGGCCAA |
| hCCR5-2 on-target_R2 | GAAGATTCCAGAGAAGAAGCC |
| hCCR5-2 off-target1_F1 | GAAAATGGCTGTTGGGTAAATC |
| hCCR5-2 off-target1_R1 | TAAGGGCCACAGACATAAAC |
| hCCR5-2 off-target1_F2 | CCAGACTATTTGGAAGATCATG |
| hCCR5-2 off-target1_R2 | TGATGAAGAAGATTCCGCCA |
| hHPRT1 on-target_F1 | ACCTTATGAAACATGAGGGC |
| hHPRT1 on-target_R1 | GGTATCTGTCACCAGTATGTATA |
| hHPRT1 on-target_F2 | GATTTTCCCACCTCACCTCT |
| hHPRT1 on-target_R2 | GTGAATATGTTAGAAAAATCTCACTG |
| hHPRT1 off-target_F1 | CATTCAATAATACCACAAAGTGAAC |
| hHPRT1 off-target_R1 | GCTGGTGTATATCCAACACTTTC |
| hHPRT1 off-target_F2 | CTTCTACTGTGCCTTCACCTC |
| hHPRT1 off-target_R2 | GAAAGCAAGCTTTGCATTATTTC |
| hRPL32P3 on-target_F1 | GAGGCCATTTCCTTTGCTTC |
| hRPL32P3 on-target_R1 | GACAAGGATTGAGAGGATTTG |
| hRPL32P3 on-target_F2 | CAAACTGTACAAGAGGAGTGG |
| hRPL32P3 on-target_R2 | CATCCTGTCTGAGACCATTG |
| hRPL32P3 off-target_F1 | CCTTTAGTACACCAAGCTCTG |
| hRPL32P3 off-target_R1 | CAACTCAATGGAGCTCCAAG |
| hRPL32P3 off-target_F2 | CTTTCTCTTTGCTCTCCAAGG |
| hRPL32P3 off-target_R2 | GAAAGTCTCCATTCTGTTCATG |
| hDNMT1_Adaptor_F | ACACTCTTTCCCTACACGACGCTCTTCCGATCTAGTGTTCAGTCTCCGTGAACGTTC |
| hDNMT1_Adaptor_R | GTGACTGGAGTTCAGACGTGTGCTCTTCCGATCTTCCTTAGCAGCTTCCTCCTCCTT |
| hCCR5-1_Adaptor_F | ACACTCTTTCCCTACACGACGCTCTTCCGATCTAACAGTTTGCATTCATGGAGGGC |
| hCCR5-1_Adaptor_R | GTGACTGGAGTTCAGACGTGTGCTCTTCCGATCTAGTTTATCAGGATGAGGATGACC |
| hFANCF_Adaptor_F | ACACTCTTTCCCTACACGACGCTCTTCCGATCTCACTACCTACGTCAGCACCTGGGACC |
| hFANCF_Adaptor_R | GTGACTGGAGTTCAGACGTGTGCTCTTCCGATCTGGAAGTTCGCTAATCCCGGAACTGGA |
| hGRIN2B_Adaptor_F | ACACTCTTTCCCTACACGACGCTCTTCCGATCTCTCTCATTCTGCAGAGCAAATACCAGAGAT |
| hGRIN2B_Adaptor_R | GTGACTGGAGTTCAGACGTGTGCTCTTCCGATCTCCTGCAAACACAAAGAAAGAGCATGTTAAA |
| hEMX1_Adaptor_F | ACACTCTTTCCCTACACGACGCTCTTCCGATCTGGCCTCCTGAGTTTCTCATCTGTGC |
| hEMX1_Adaptor_R | GTGACTGGAGTTCAGACGTGTGCTCTTCCGATCTCCTGCTTCGTGGCAATGCGCCAC |
| hCCR5-2 on-target_Adaptor_F | ACACTCTTTCCCTACACGACGCTCTTCCGATCTCCTGCCAAAAAATCAATGTGAAG |
| hCCR5-2 on-target_Adaptor_R | GTGACTGGAGTTCAGACGTGTGCTCTTCCGATCTGAAGGGGACAGTAAGAAGGAA |
| hCCR5-2 off-target_Adaptor_F | ACACTCTTTCCCTACACGACGCTCTTCCGATCTTCCCTGTCATAAATTTGACGTG |
| hCCR5-2 off-target_Adaptor_R | GTGACTGGAGTTCAGACGTGTGCTCTTCCGATCTGAAAAAGCAGATCAGAGATGGC |

†Sequences of the forward and reverse adapter primers used in next-generation sequencing are shown in green and blue, respectively.

**Table S3. Sequence information for the sgRNA for dead and nickase (D10A) SpCas9 used in this study.**

| Target gene  (guide No.) | CRISPR-Cas9  target sequence (5′-3′) | sgRNA sequence for dead or nickase SpCas9 (5′-3′) |
| --- | --- | --- |
| hDNMT1 **sgRNA1** | **TTAACAGCTGACCCAATAAGTGG** | **5’GUUAACAGCUGACCCAAUAAGGUUUUAGAGCUAGAAAUAGCAAGUUAAAAUAAGGCUAGUCCGUUAUCAACUUGAAAAAGUGGCACCGAGUCGGUGC** 3’ |
| hCCR5-2 **sgRNA2** | **AACACCAGTGAGTAGAGCGGAGG** | **5’GAACACCAGUGAGUAGAGCGGGUUUUAGAGCUAGAAAUAGCAAGUUAAAAUAAGGCUAGUCCGUUAUCAACUUGAAAAAGUGGCACCGAGUCGGUGC** 3’ |

†PAM sequence (NGG) in the target DNA for the SpCas9 effector is shown in blue. Underlined sequence in sgRNA indicates the target sequence.

**Supplementary Figures**


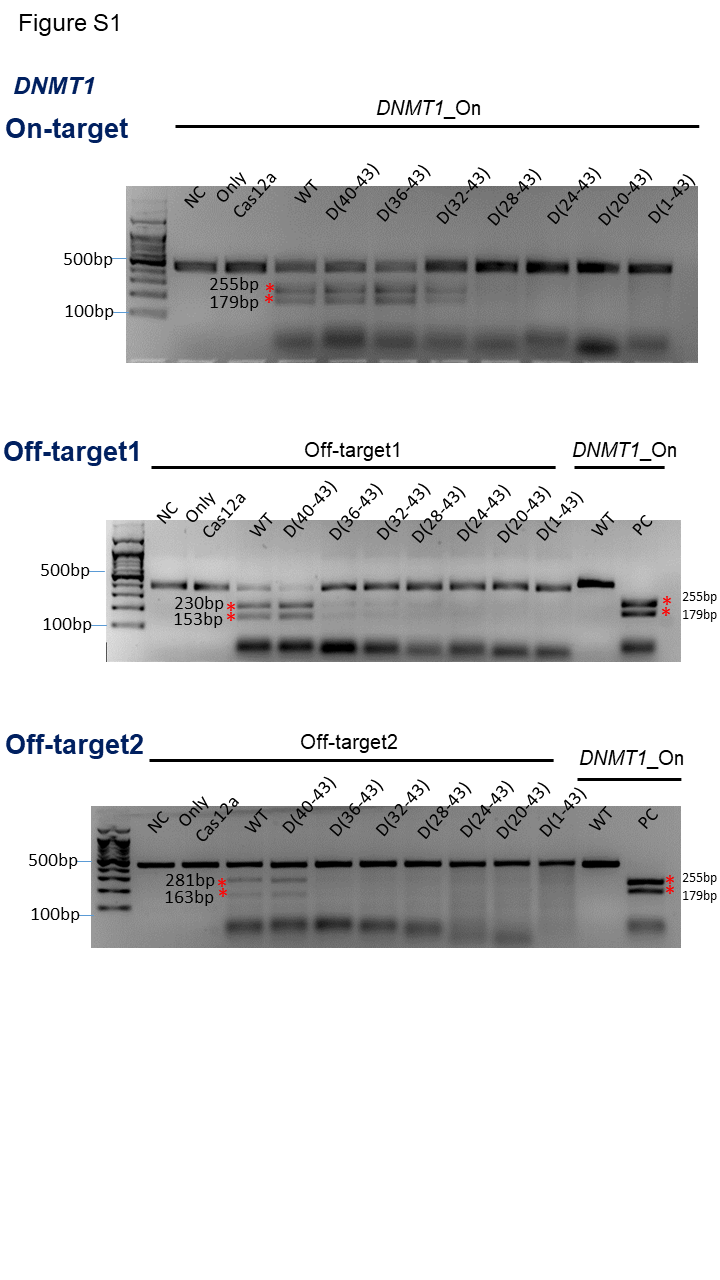


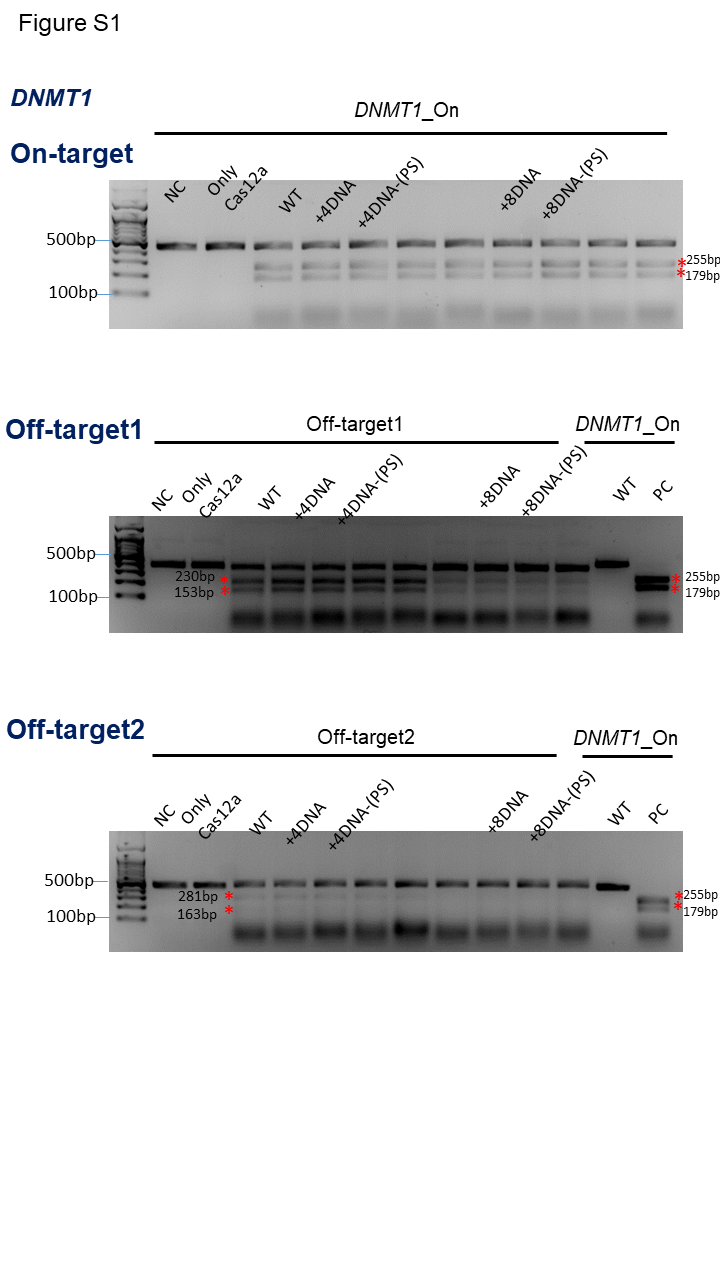


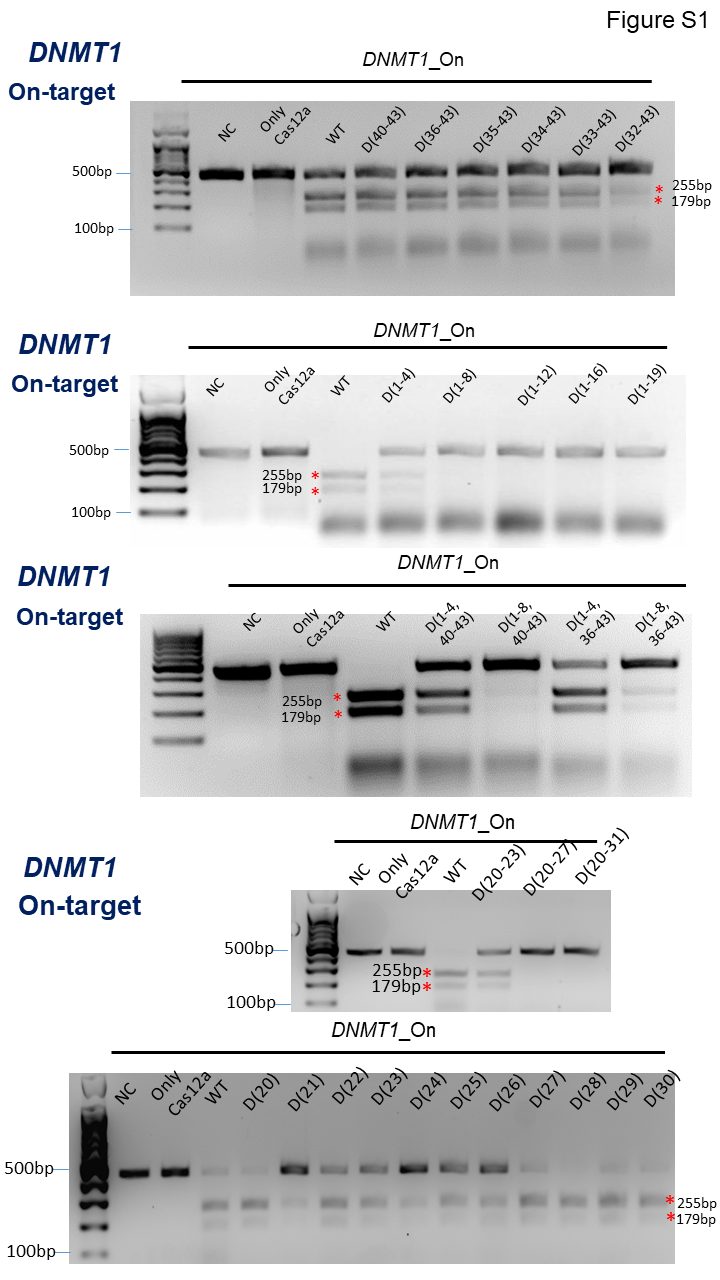


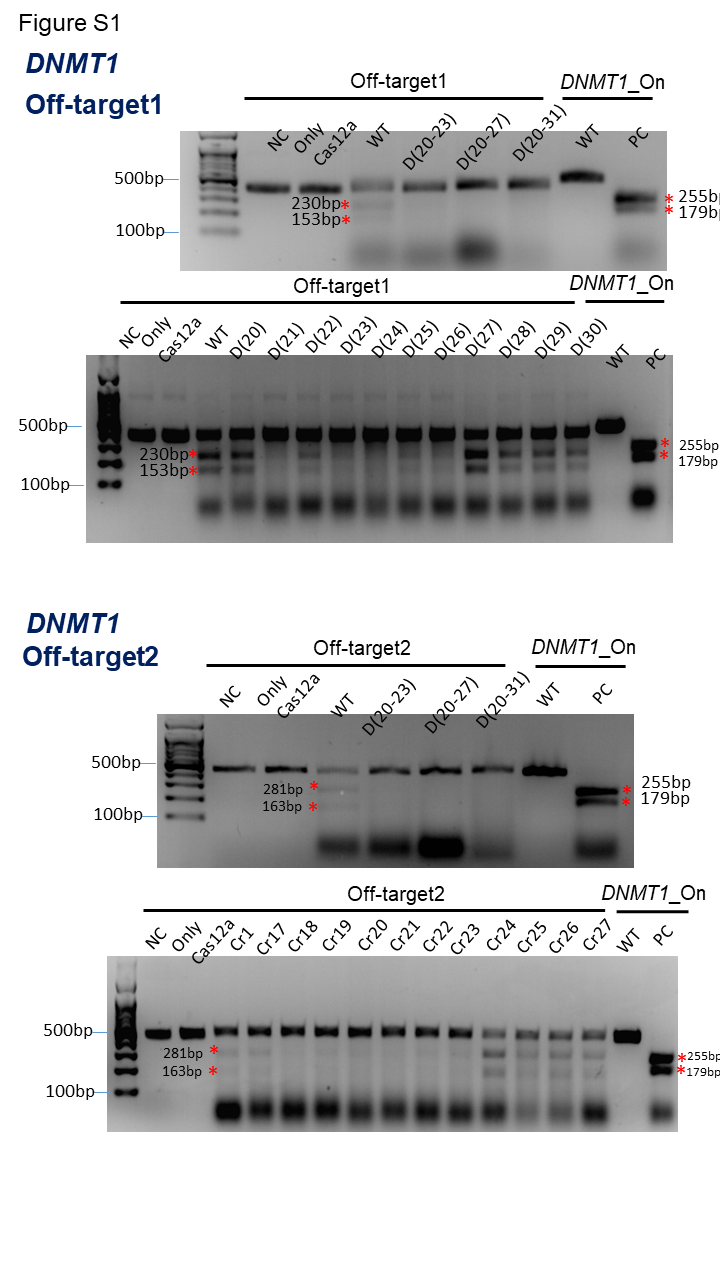


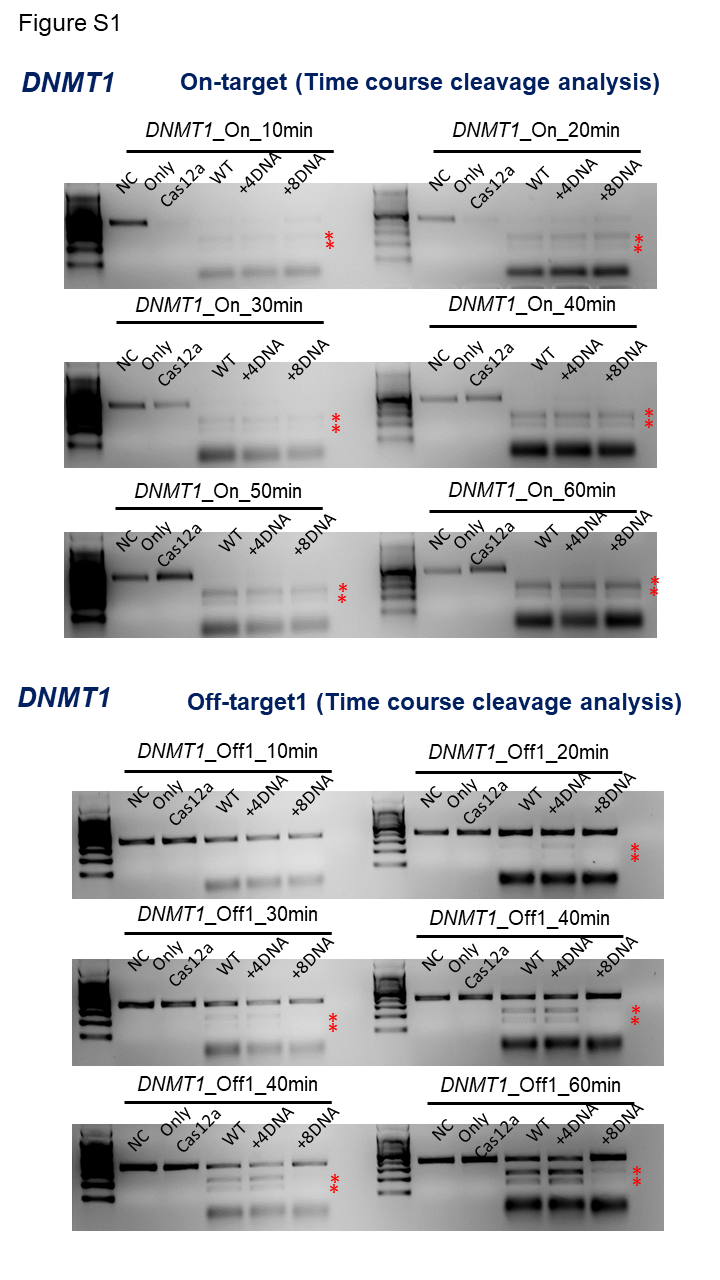


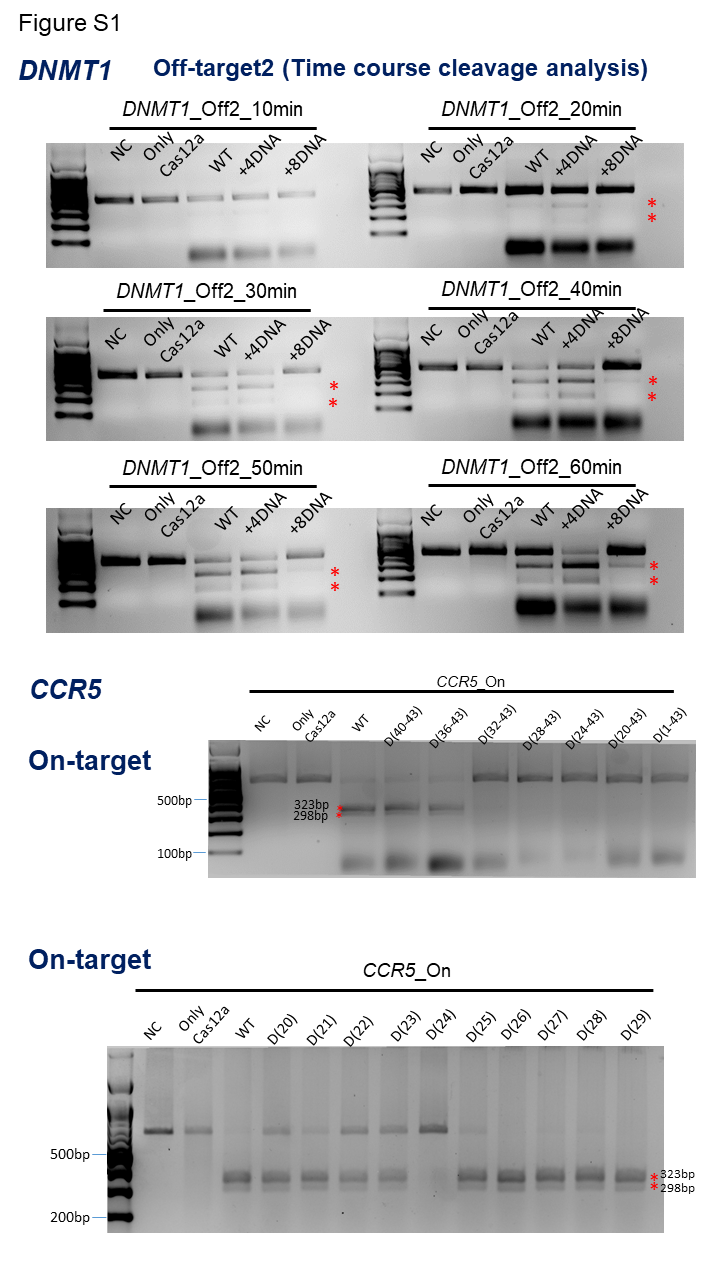


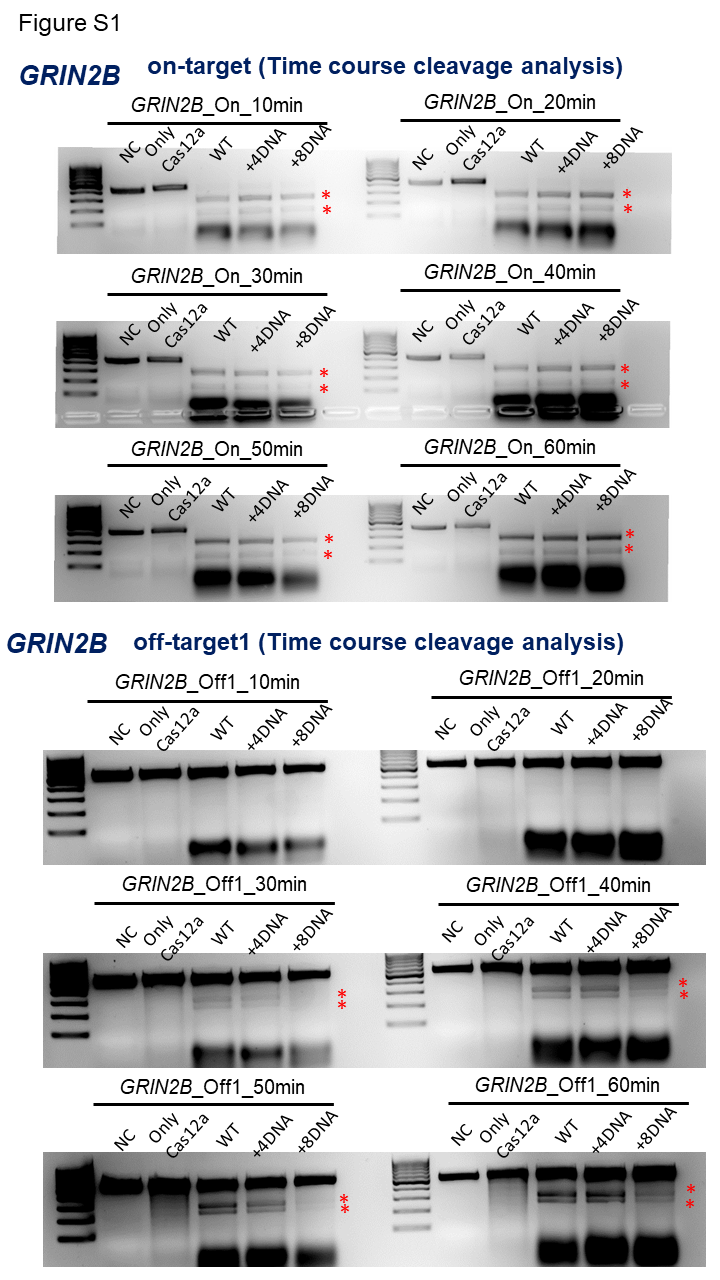


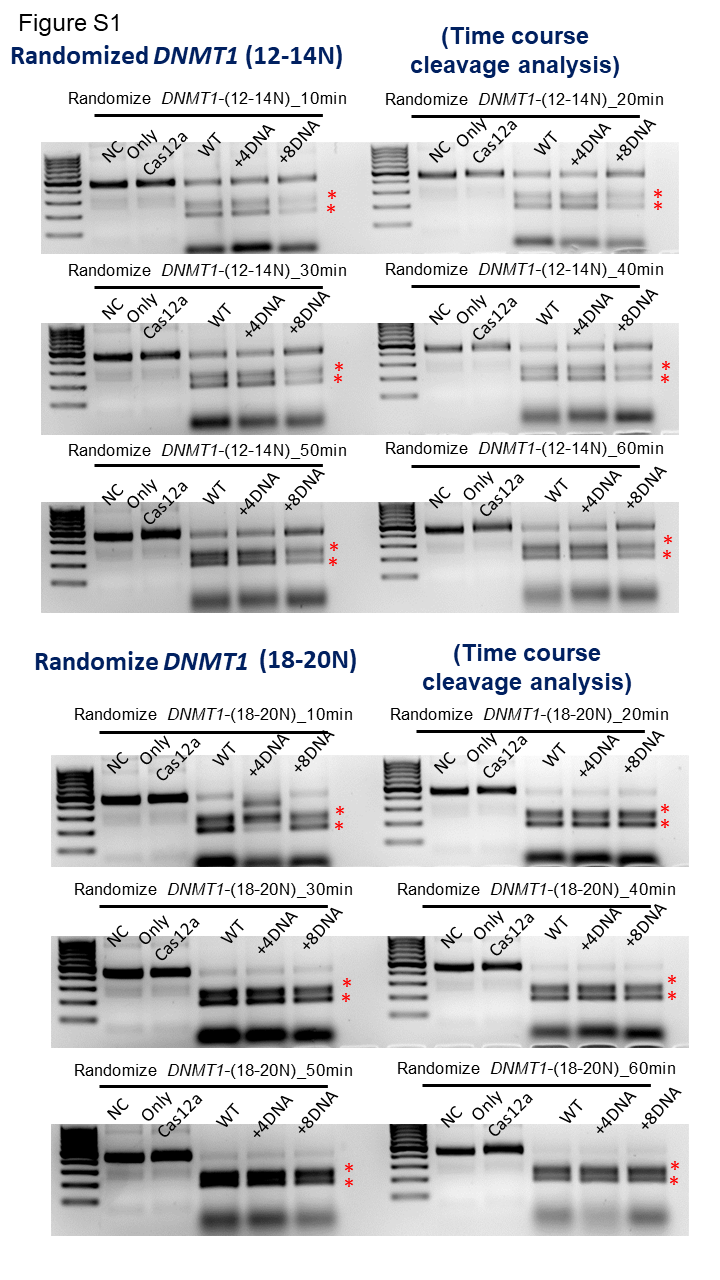


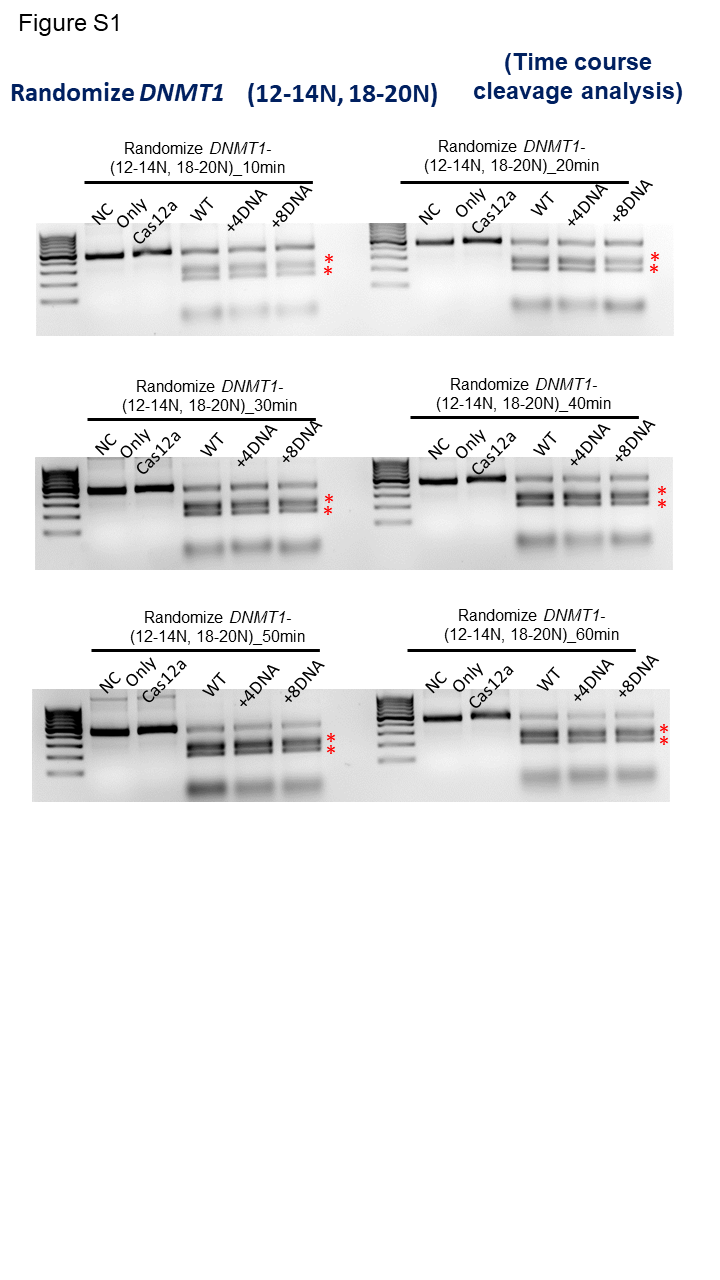


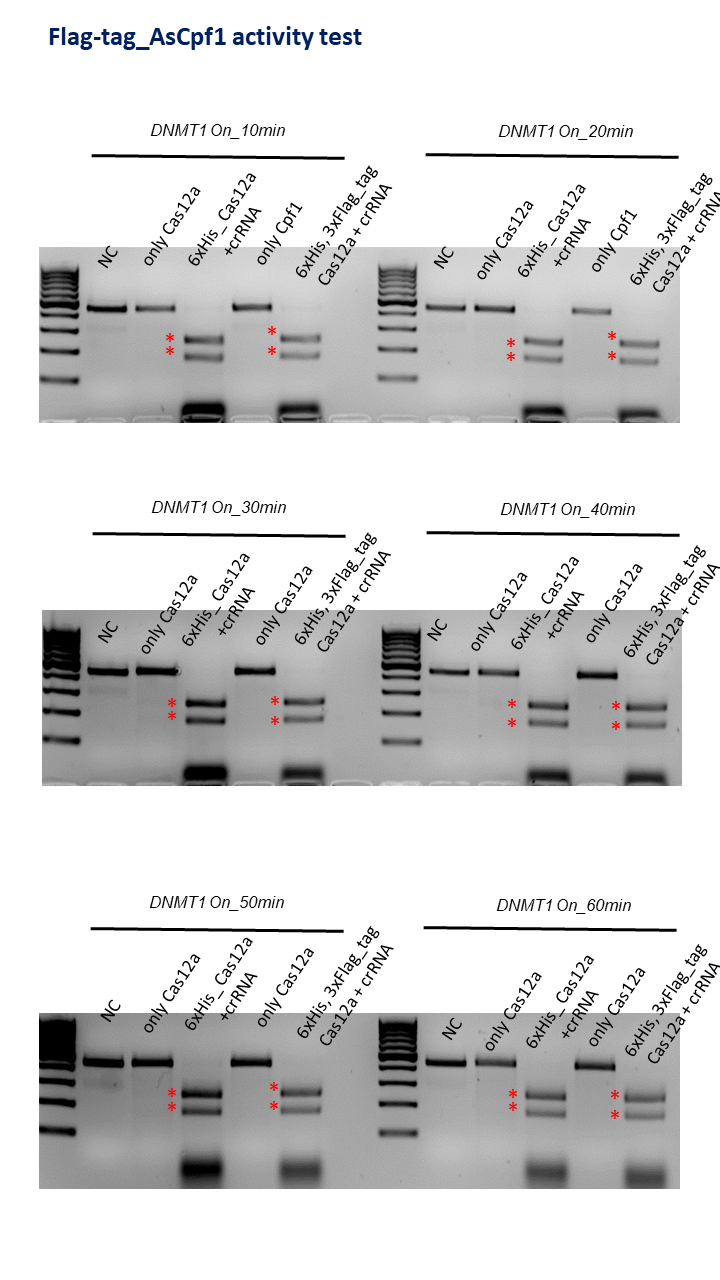


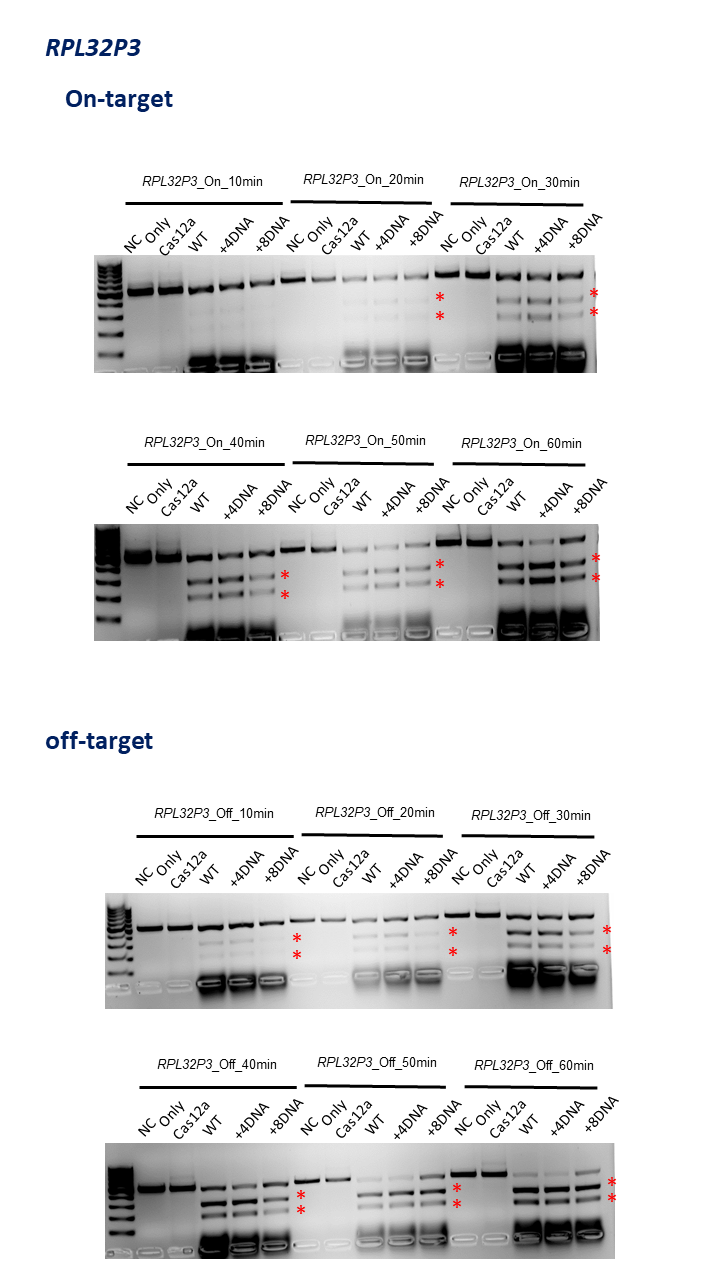


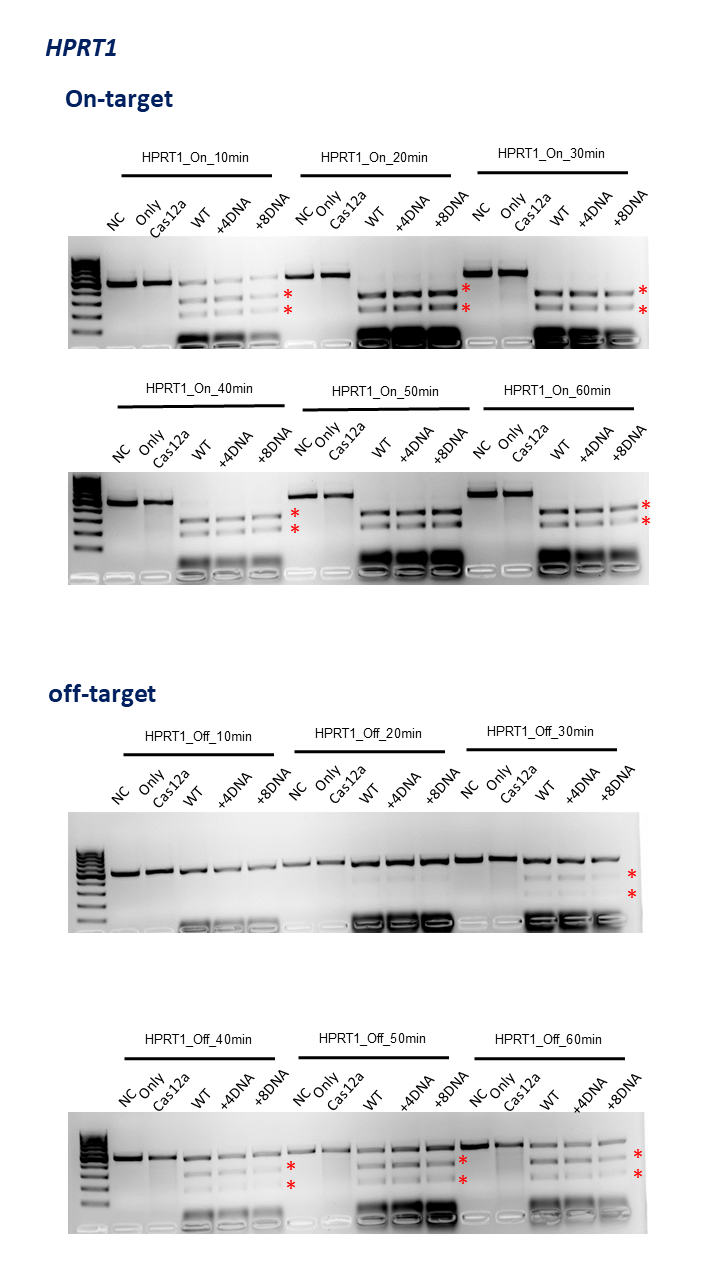


**[Figure S1] Result of *in-vitro* on/off-target DNA amplicon cleavage assay for various target gene sequences.** PCR amplicons were obtained from purified genomic DNA (HEK293FT) using DNA primers **(Table S2)** corresponding to each target locus (*DNMT1, CCR5, FANCF, GRIN2B, EMX1, HPRT1, RPL32P3*). Each amplicon was cleaved and separated on 2% agarose gel. Cleaved DNA fragments are indicated by red asterisks. NC: negative control, Only Cas12a: Only protein treated, WT: Wild-type crRNA was treated with Cas12a, +4 DNA: Chimeric crRNA (sequential 4-nt DNA substitution at 3'-end of crRNA) was treated with Cas12a, +8 DNA: Chimeric crRNA (sequential 8-nt DNA substitution at 3'-end of crRNA) was treated with Cas12a.


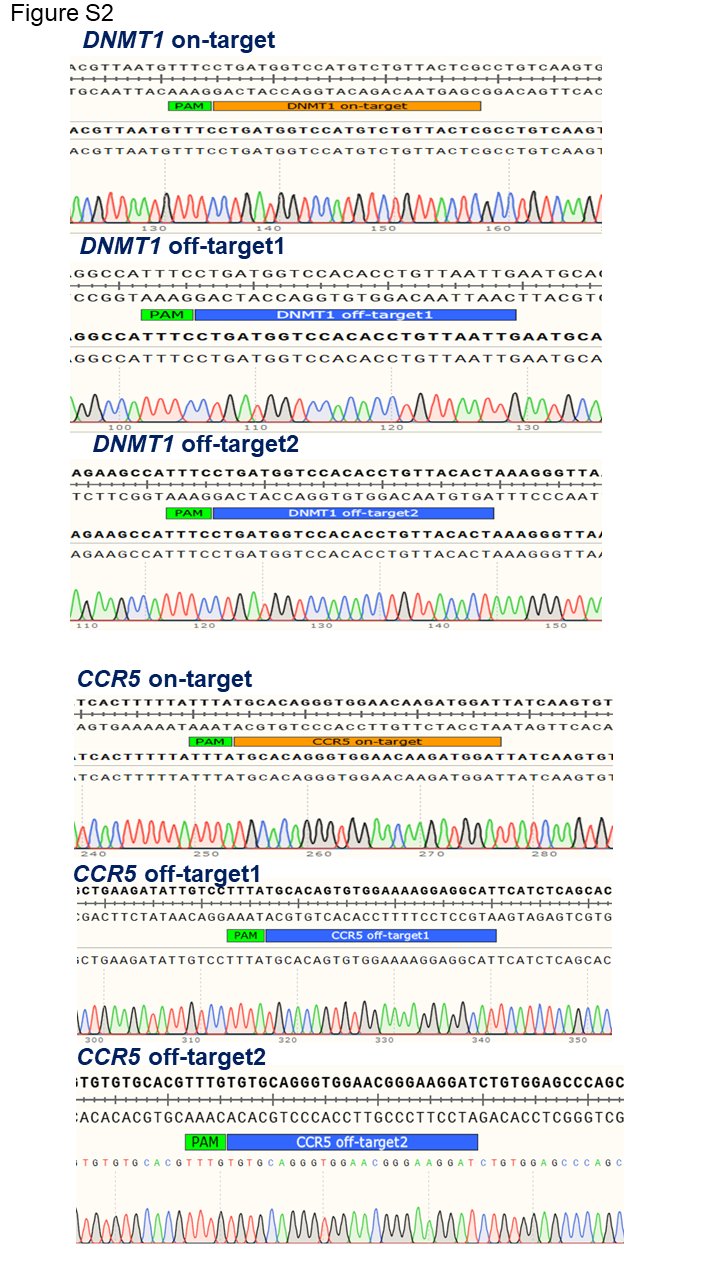


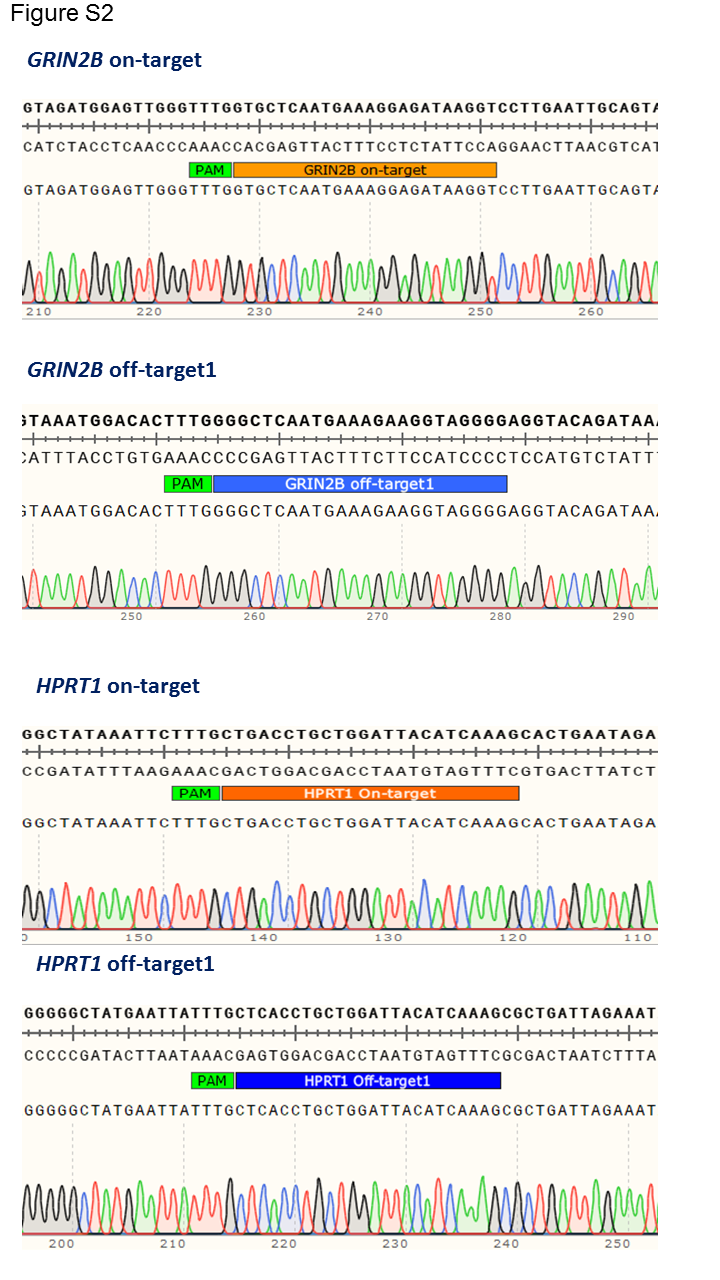


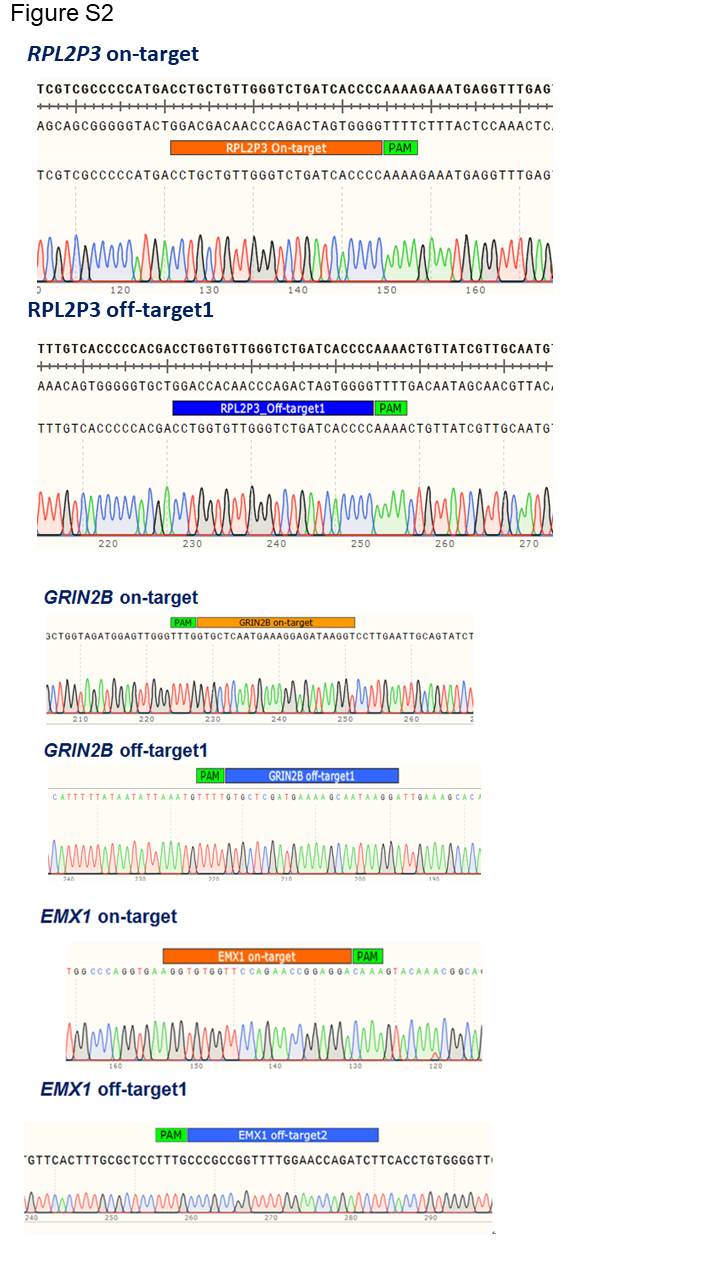


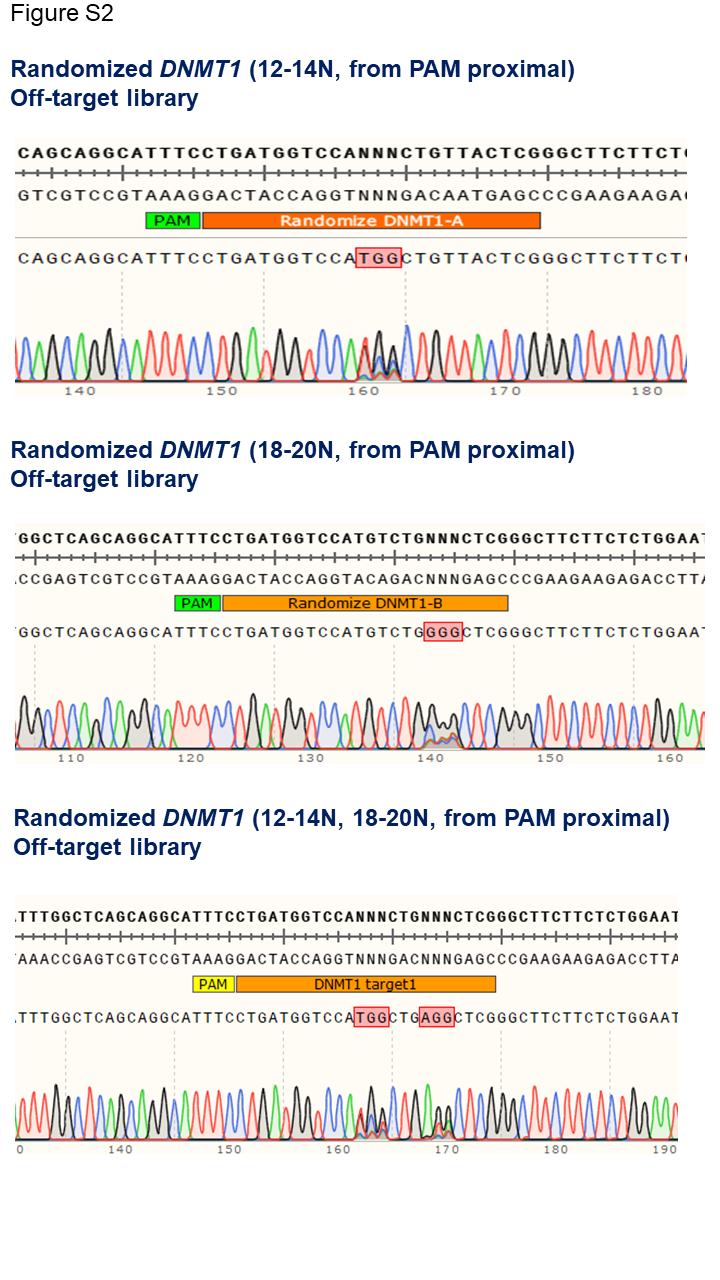


**[Figure S2] Sanger sequencing data from each PCR amplicon of on-target and predicted off-target sites.** PAM sequence (TTTN) for AsCas12a and on- or off-target sequence in each gene was shown in green, orange and blue color, respectively.


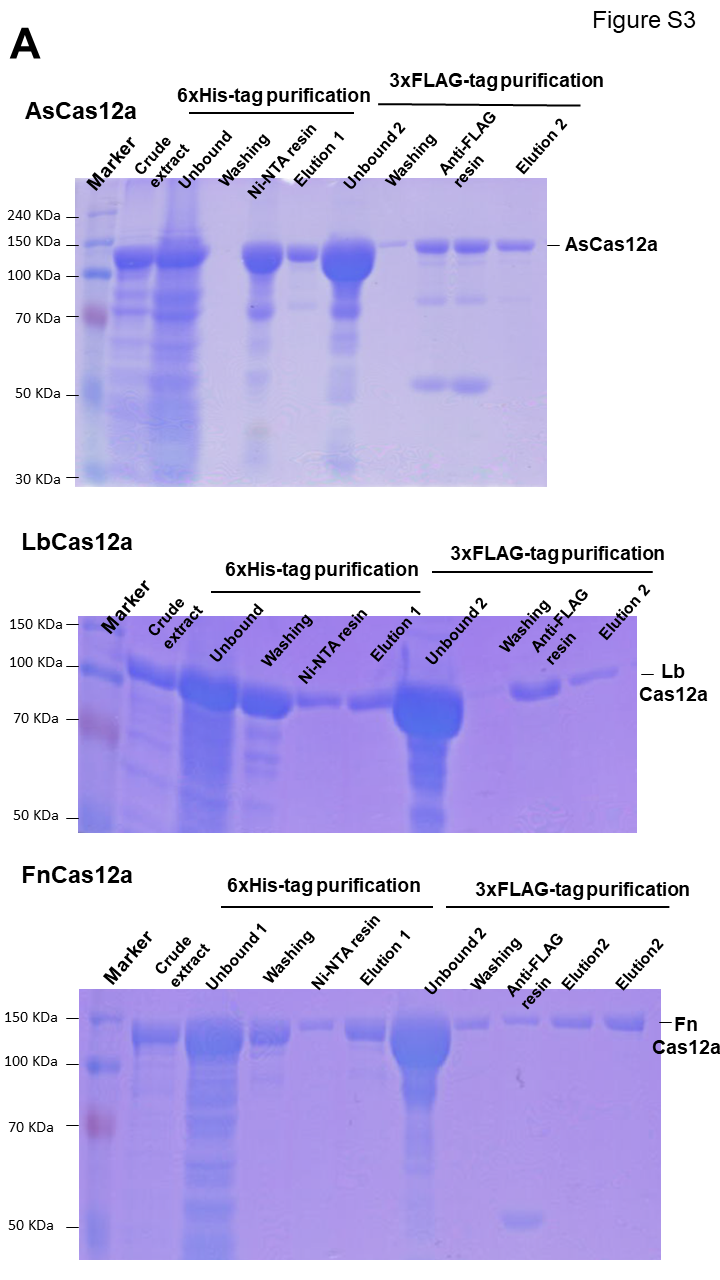


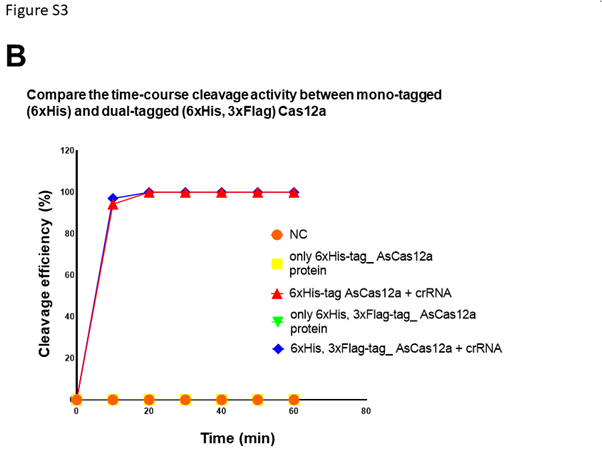


**[Figure S3] Purification of the recombinant CRISPR-Cas12a orthologue proteins using affinity chromatography. (A)** Purity of eluted recombinant protein was confirmed by 10% SDS-PAGE after sequential loading on affinity chromatography using 6xHis-tag and 3xFLAG-tag connected to each N, C-terminus of As, Lb, FnCas12a proteins. **(B)** The target DNA amplicon was cleaved and analyzed for endonuclease activity using purified Cas12a proteins with 6xHis-tag single purification or sequential purification using dual 6xHis-tag and 3xFLAG-tag.


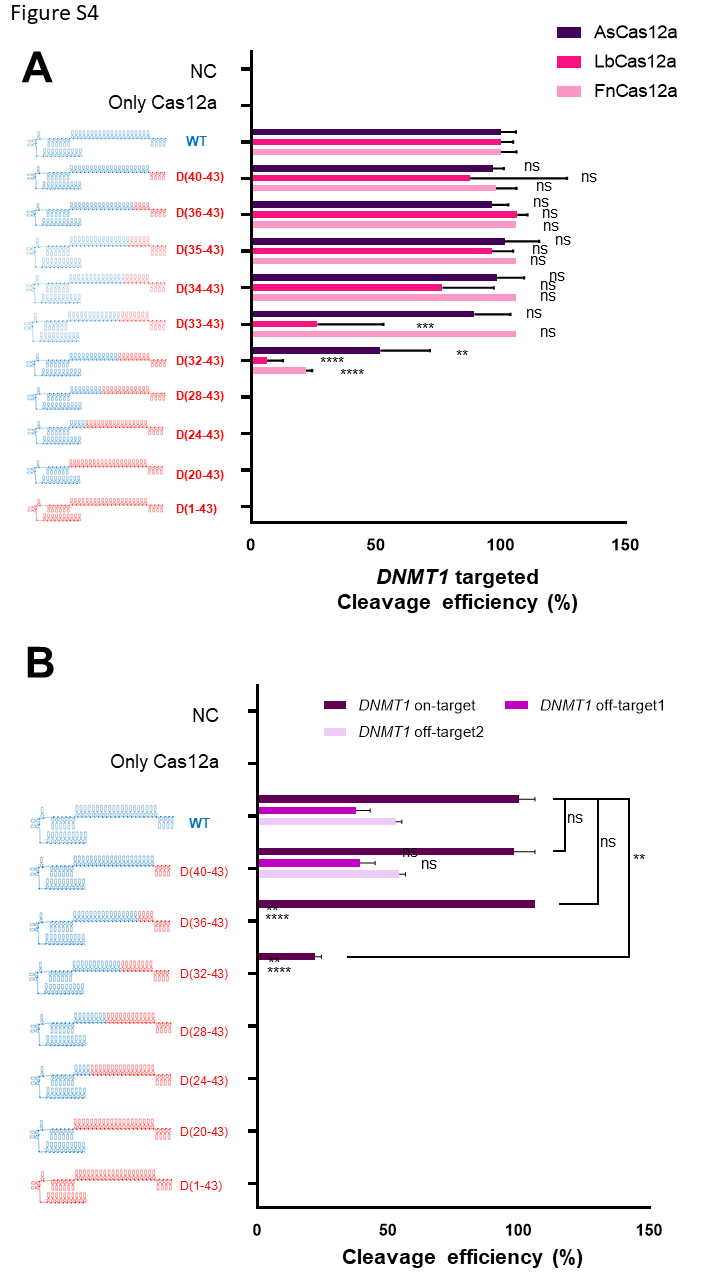


**[Figure S4] Target DNA cleavage by CRISPR-Cas12a orthologues using chimeric DNA-RNA guides. (A)** Comparison for AsCas12a, LbCas12a and FnCas12a cleavage efficiency for *DNMT1* amplicon by using chimeric (cr)RNA. (cr)RNA of AsCas12a was partially replaced with DNA from 3’-end. The RNA portion of the (cr)RNA is shown in blue, and the DNA portion is shown in red (number of substituted DNA is indicated). Cleavage efficiency (%) is a relative ratio that is normalized to wt-(cr)RNA and shown in dark brown (AsCas12a), dark pink (LbCas12a) and pink (FnCas12a) color. All cleavage efficiencies were calculated from agarose gel separated band intensity (cleavage efficiency (%) = cleaved fragment intensity / total fragment intensity) and normalized to wild-type (cr)RNA. Data are shown as means ± s.e.m. from three independent experiments. *P*-values are calculated using a one way ANOVA with Tukey’s test (ns: not significant, *P**:<0.0332, *P***:<0.0021, *P****:<0.0002, *P*****:<0.0001). **(B)** Off-target cleavage activity of chimeric (cr)RNA guided FnCas12a. On**/**off-target DNA (*DNMT1*: on-target (dark brown), off-target1 (pink), off-target2 (purple)) amplicon cleavage was performed to confirm the target specificity of FnCas12a using a chimeric DNA-RNA guide (serial 4nt DNA substitution of (cr)RNA from 3′-end). All the cleavage efficiency was calculated from agarose gel separated band intensity (cleavage efficiency (%) = cleaved fragment intensity / total fragment intensity) and normalized to wild-type (cr)RNA. Data are shown as means ± s.e.m. from three independent experiments. *P*-values are calculated using a two-tailed Student′s t-test (ns: not significant, P*:<0.05, P**:<0.01, P***:<0.001, P****:<0.0001).


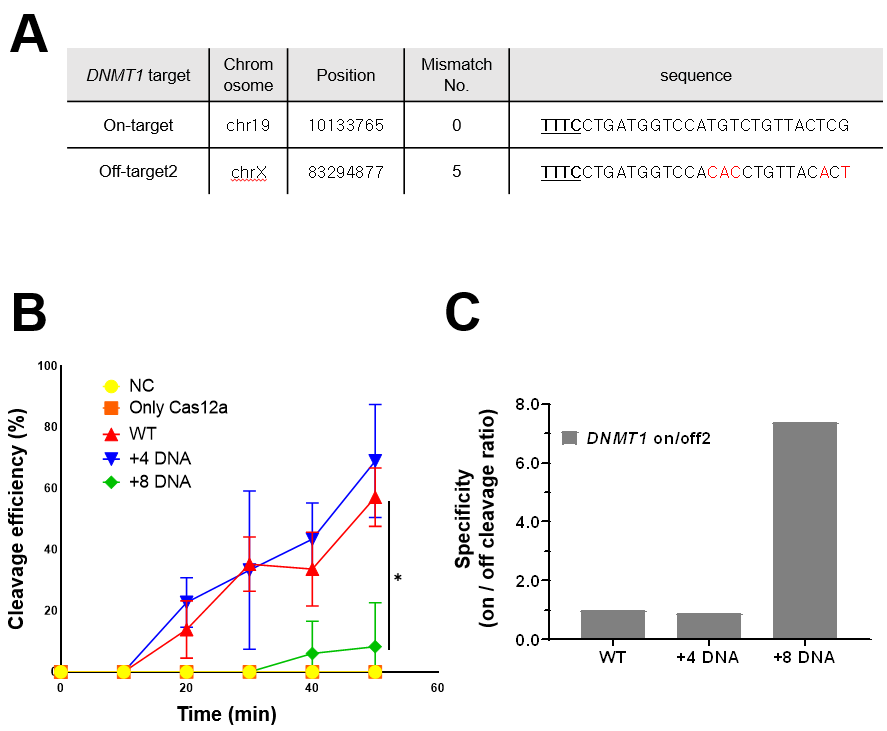


**[Figure S5] Time-course cleavage analysis for *DNMT1* off-target2 using chimeric DNA-RNA guided AsCas12a. (A)** Off-target2 DNA amplicon, which has 5 mismatch nucleotides, was prepared to confirm the target specificity of AsCas12a guided by chimeric DNA-RNA (sequential 4-nt and 8-nt DNA substitutions in the 3′-end of (cr)RNA). **(B)** The chimeric DNA-RNA guided Cas12a cleavage activity on off-target2 was measured as time-course analysis. The cleavage efficiency was calculated from agarose gel separated band intensity (cleaved fragment intensity (%) / total fragment intensity (%)) for 50 minutes at 10min interval points and normalized to wild-type (cr)RNA. Data are shown as means ± s.e.m. from three independent experiments. *P*-values are calculated using a two-tailed Student’s t-test (ns: not significant, P*:<0.05, P**:<0.01, P***:<0.001, P****:<0.0001). NC: negative control, only Cas12a: only protein treated, WT: Wild-type crRNA was treated with Cas12a, +4 DNA: Chimeric crRNA (sequential 4-nt DNA substitution at 3’-end of crRNA) was treated with Cas12a, +8 DNA: Chimeric crRNA (sequential 8-nt DNA substitution at 3’-end of crRNA) was treated with Cas12a. **(C)** Target specificity (on/off cleavage ratio (%)) of AsCas12a using chimeric DNA-RNA guides, calculated from (B).


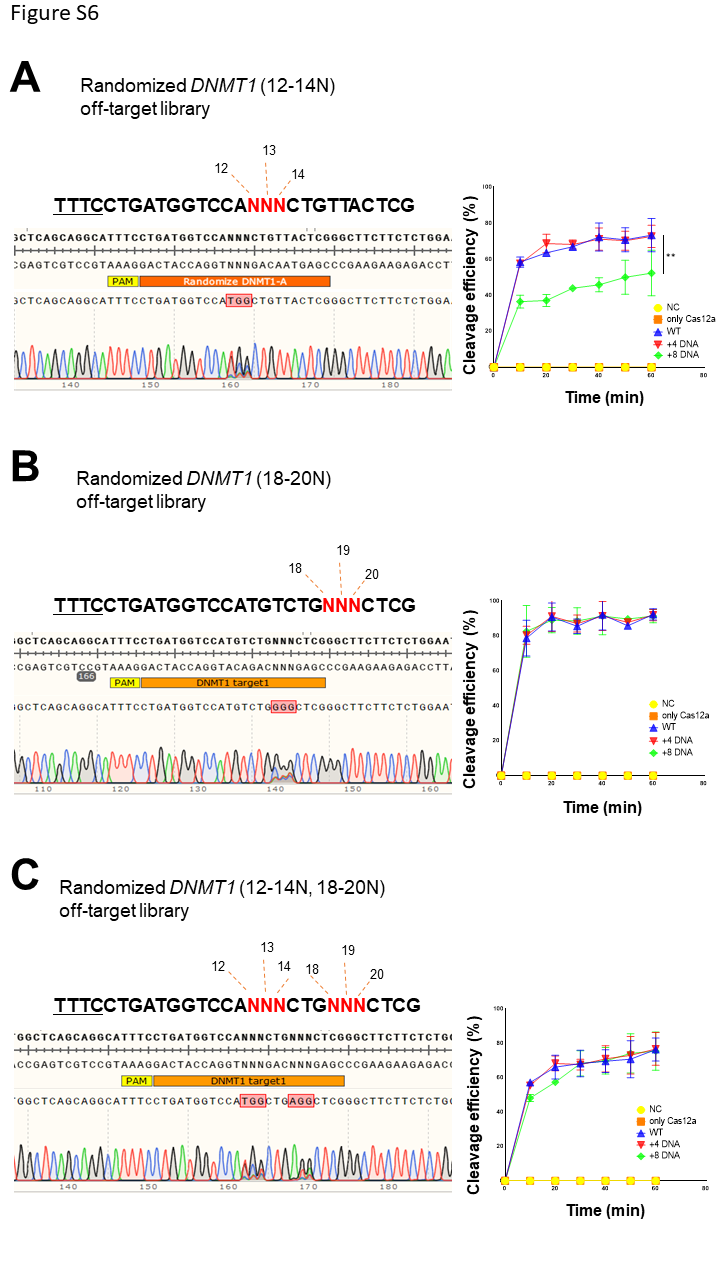


**[Figure S6] Time-course cleavage analysis for randomized *DNMT1* library using chimeric DNA-RNA guided AsCas12a.** Time-course cleavage of chimeric DNA-RNA guided Cas12a against randomized *DNMT1* targets, which is prepared by T-vector cloning. Cleavage was performed for (12-14N) library **(A)**, (18-20N) library **(B)**, (12-14N, 18-20N) library **(C)** of *DNMT1* targets for 60 minutes at 10-minute intervals. 12-14N in the random library means that the 3 bases of the position 12-14 nt from PAM (TTTC) in the protospacer is randomly mutated. 18-20N: random mutation of 3 based at position 18-20 nt from PAM in protospacer. 12-14N, 18-20N: random mutation of 6 bases at position 12-14, 18-20 nt from PAM in protospacer. All data measurement and analysis proceeded the same as in **(Figure 4)**. NC: negative control, only Cas12a: only protein treated, WT: Wild-type crRNA was treated with Cas12a, +4 DNA: Chimeric crRNA (sequential 4-nt DNA substitution at 3’-end of crRNA) was treated with Cas12a, +8 DNA: Chimeric crRNA (sequential 8-nt DNA substitution at 3’-end of crRNA) was treated with Cas12a.


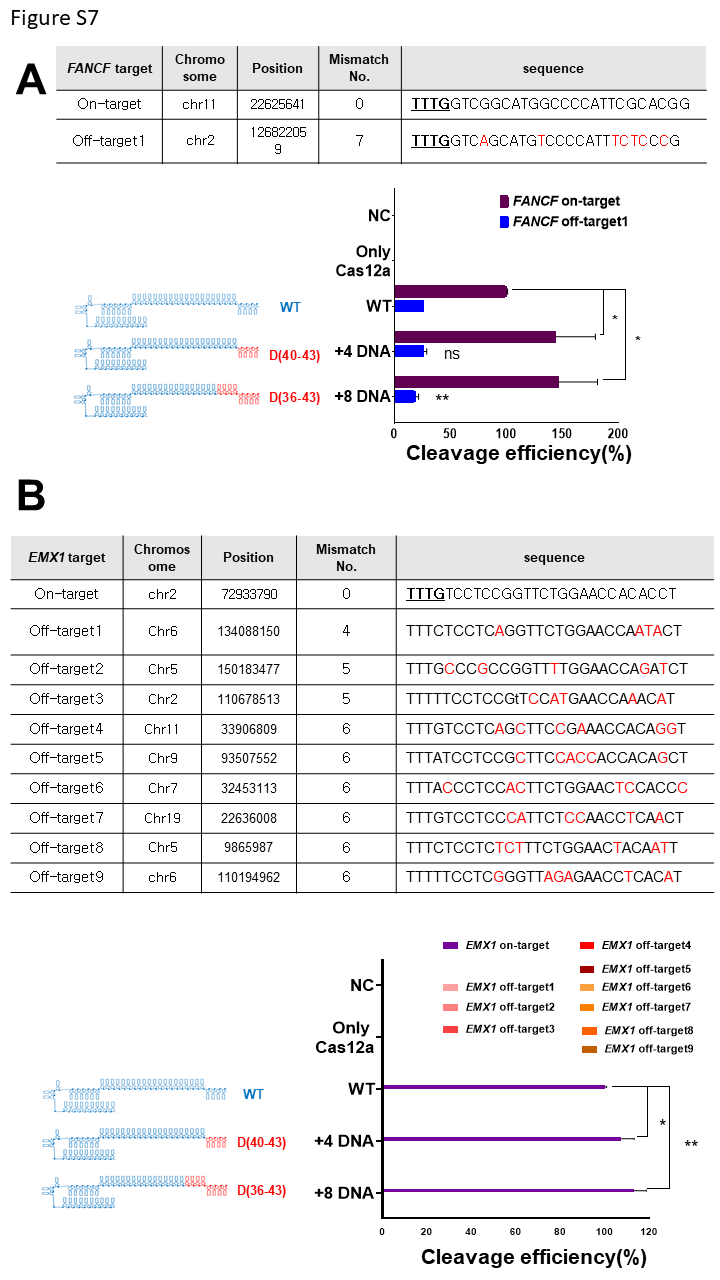


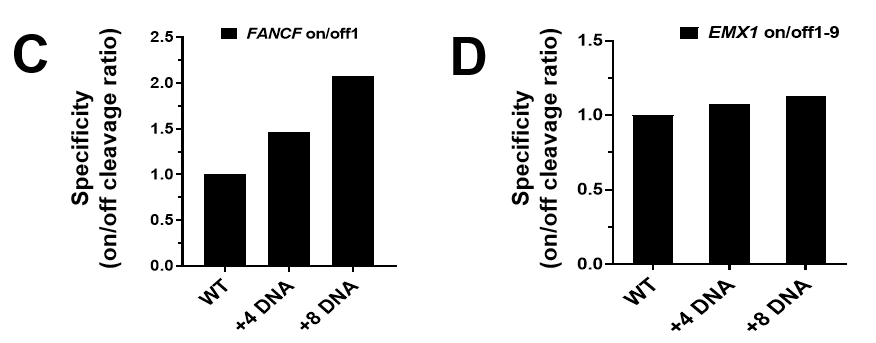


[Figure S7] Off-target cleavage assay of Cas12a using chimeric DNA-RNA guides. (A) Cleavage efficiency of the target (dark brown) and off-target (off1: blue) of *FANCF* DNA using chimeric DNA-RNA guided (3′-end 4-nt, 8-nt DNA substitutions) AsCas12a. (B) Cleavage efficiency of the on- / off-target of *EMX1* DNA using chimeric DNA-RNA guided (3′-end 4-nt, 8-nt DNA substitutions) AsCas12a. All cleavage efficiencies were calculated from agarose gel separated band intensity (cleaved fragment intensity (%) / total fragment intensity (%)) and normalized to wild-type (cr)RNA. Data are shown as means ± s.e.m. from three independent experiments. *P*-values are calculated using a two-tailed Student′s t-test (ns: not significant, P*:<0.05, P**:<0.01, P***:<0.001, P****:<0.0001). On- and off-target sequence information for each gene is shown at the top. The PAM sequence is underlined and shown in bold. Mismatch sequences to wild-type reference are shown in red. (C, D) Comparison of *FANCF* (C) and *EMX1* (D) target specificities of AsCas12a using chimeric DNA-RNA guides with 4-, 8-nt DNA substitutions from the 3′-end of the (cr)RNA). Target specificity is shown in black (on/off 1). The number of substituted DNA nucleotides in AsCas12a (cr)RNA used for each gene targeting is indicated by red color. NC: negative control, only Cas12a: only protein treated, WT: wild-type (cr)RNA, +4 DNA: 3'-end 4-nt DNA substituted (cr)RNA, +8 DNA: 3′-end 8-nt DNA substituted (cr)RNA.


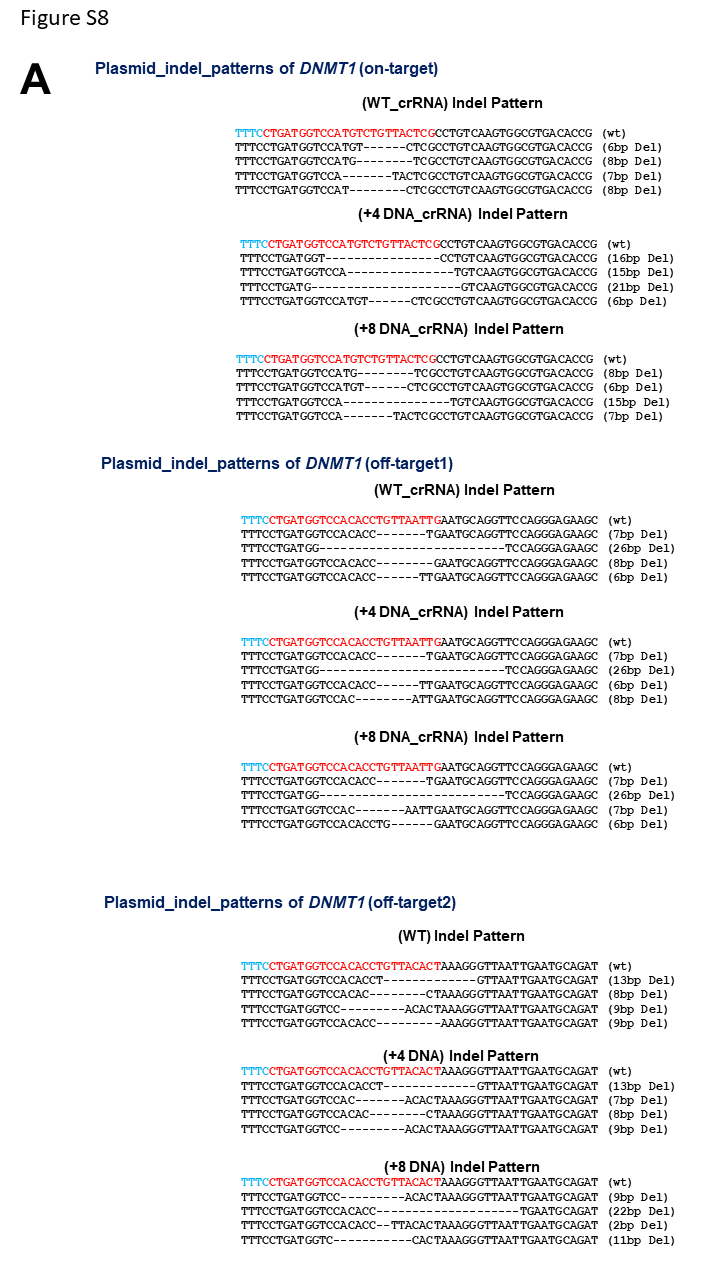


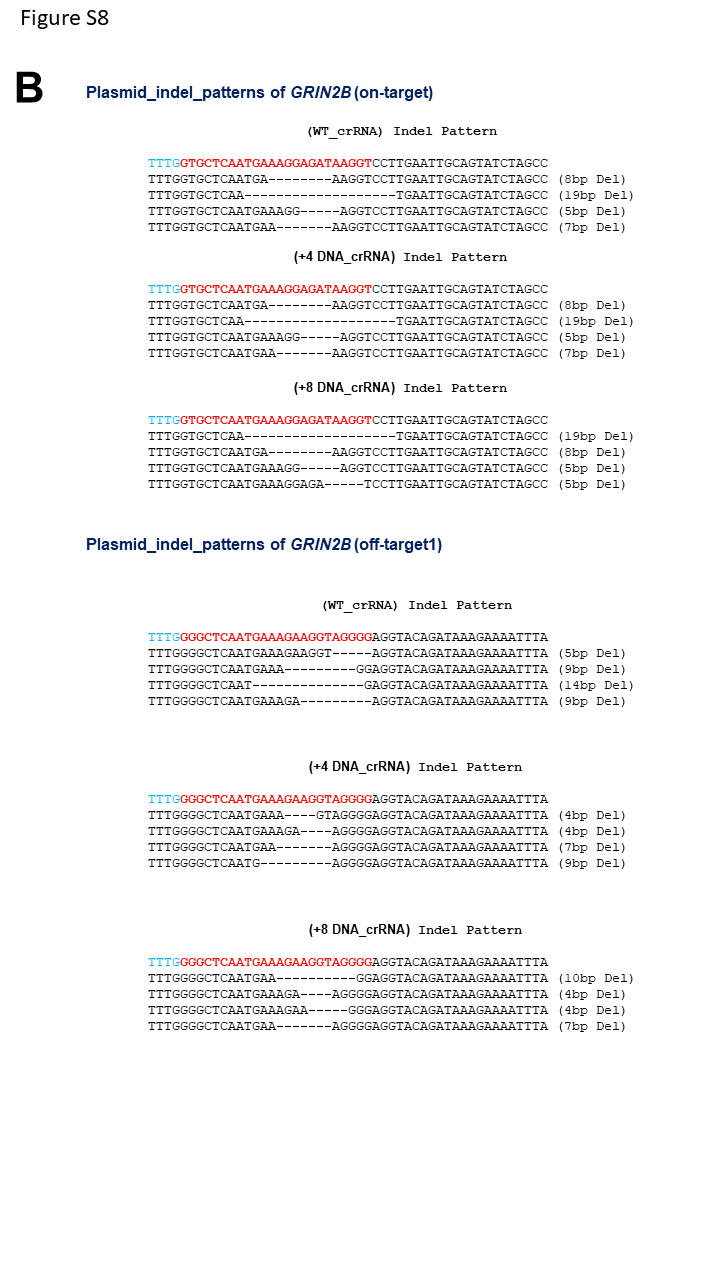


**[Figure S8] Representative indel patterns from plasmid (*DNMT1*, *GRIN2B*) targeted genome editing by chimeric (cr)RNA guided AsCas12a.** Analyzed indel pattern from *DNMT1* (A) and *GRIN2B* (B) targeted amplicon data (NGS) of chimeric (cr)RNA guided genome editing in HEK293FT cell. PAM sequence (TTTN) for AsCas12a is shown in blue and target sequence is shown in red. Deleted sequence relative to the wild-type reference sequence is indicated by the dashed line. WT_crRNA: Wild-type crRNA was treated with Cas12a, +4 DNA_crRNA: Chimeric crRNA (sequential 4-nt DNA substitution at 3'-end of crRNA) was treated with Cas12a, +8 DNA_crRNA: Chimeric crRNA (sequential 8-nt DNA substitution at 3'-end of crRNA) was treated with Cas12a.


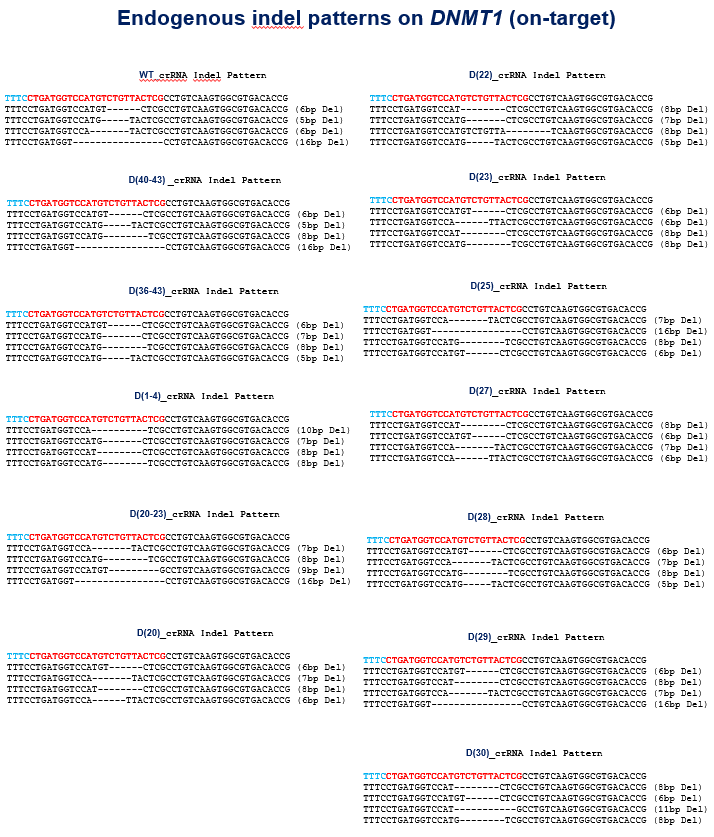


**[Figure S9] Representative indel patterns from endogenous locus (*DNMT1*) targeted genome editing by using various chimeric (cr)RNA guided AsCas12a.** Analyzed indel pattern from NGS data of chimeric (cr)RNA guided genome editing in HEK293FT cell. PAM sequence (TTTN) for AsCas12a is shown in blue and target sequence is shown in red. Deleted sequence relative to the wild-type reference sequence is indicated by the dashed line. WT_crRNA: Wild-type crRNA was treated with Cas12a. 'D' indicates a DNA and the position number of substituted DNA nucleotides in (cr)RNA is indicated as in Figure 1A.


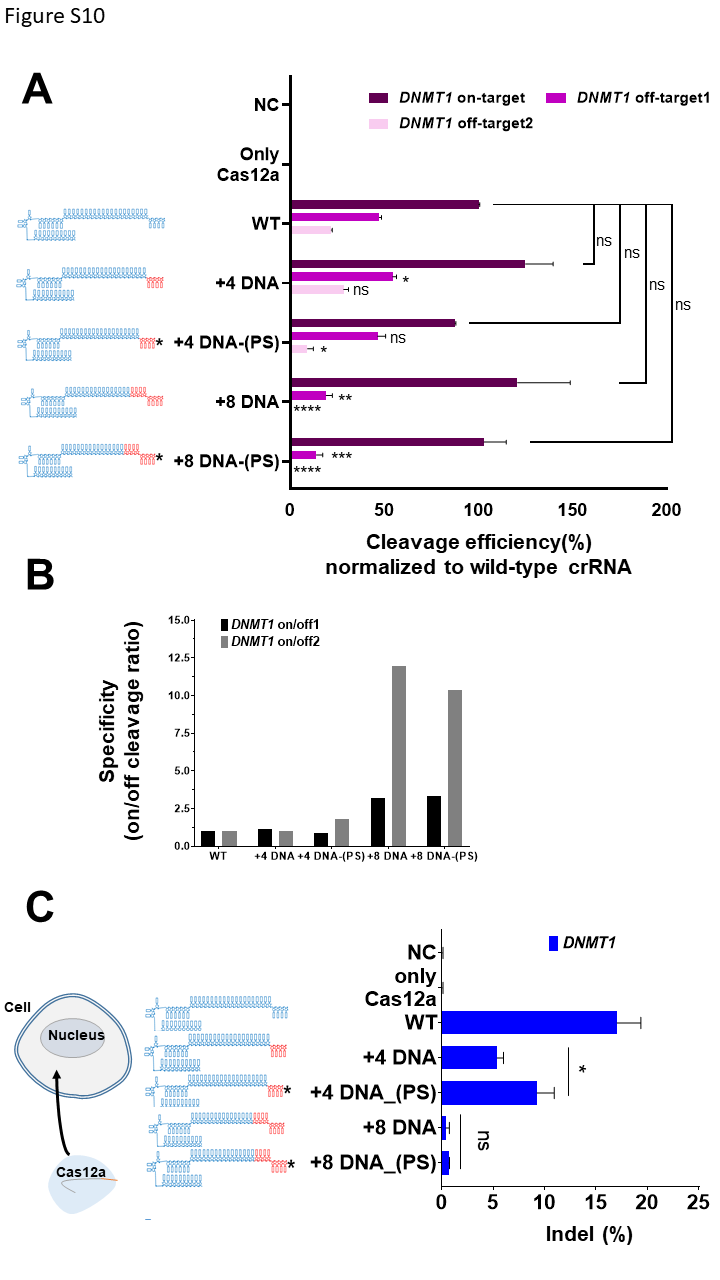


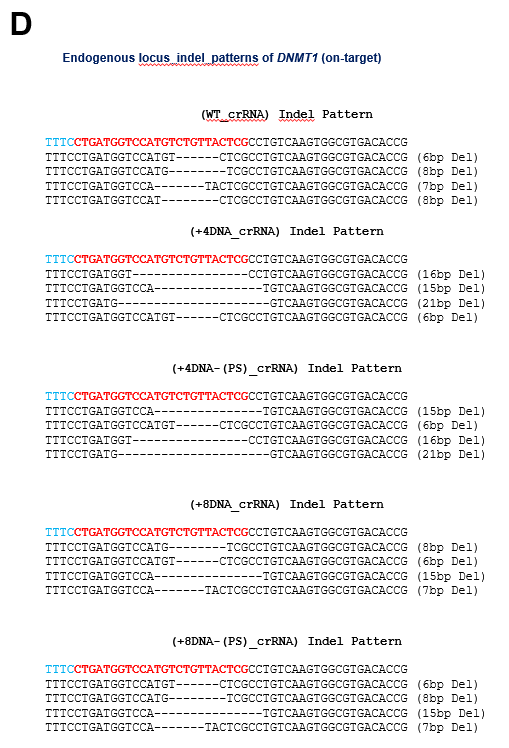


[Figure S10] Intracellular genome editing of Cas12a using 3′-end chemically modified chimeric DNA-RNA guides. (A) *DNMT1* on/off-target amplicon cleavage by AsCas12a using chimeric DNA-RNA guides with 3'-end 4-nt or 8-nt DNA substitution and 3'-end PS modification. The relative cleavage efficiency (%) of on-target (dark brown), off-target 1 (dark pink) and off-target 2 (light pink) are indicated by different colors. The RNA nucleotide of the Cas12a (cr)RNA is shown in blue, and the substituted DNA nucleotide is shown in red. Asterisk indicates the 3’-end PS modification of (cr)RNA. All the cleavage efficiency was calculated from agarose gel separated band intensity (cleavage efficiency (%) = cleaved fragment intensity / total fragment intensity) and normalized to wild-type (cr)RNA. Data are shown as means ± s.e.m. from three independent experiments. *P*-values are calculated using a two-tailed Student′s t-test (ns: not significant, P*:<0.05, P**:<0.01, P***:<0.001, P****:<0.0001). (B) Determination of target specificity (on/off cleavage ratio (%)) calculated from (A) of the chimeric DNA-RNA guide with 3′-end PS modification. Target specificity is shown in black (On/Off 1) and dark gray (On/Off 2). (C) Intracellular *DNMT1* editing using 3′-end PS-modified chimeric DNA-RNA guides. Indel ratio (%) is calculated by targeted amplicon sequencing from *DNMT1* site in HEK293FT cells (indel frequency (%) = mutant DNA read number / total DNA read number) and normalized to wild-type (cr)RNA. Data are shown as means ± s.e.m. from three independent experiments. *P*-values are calculated using a two-tailed Student’s t-test (ns: not significant, P*:<0.05, P**:<0.01, P***:<0.001, P****:<0.0001). (D) Representative indel pattern from 3'-end PS modified chimeric (cr)RNA guided genome editing of Cas12a in HEK293FT cell. PAM sequence (TTTN) for AsCas12a is shown in cyan and target sequence is shown in red. Deleted sequence relative to the wild-type reference sequence is indicated by the dashed line. NC: negative control, only Cas12a: only protein treated, WT_crRNA: Wild-type crRNA was treated with Cas12a, +4 DNA_crRNA: Chimeric crRNA (sequential 4-nt DNA substitution at 3'-end of crRNA) was treated with Cas12a, +8 DNA_crRNA: Chimeric crRNA (sequential 8-nt DNA substitution at 3'-end of crRNA) was treated with Cas12a. PS indicates phosphorothioate modification at 3’-end of crRNA.


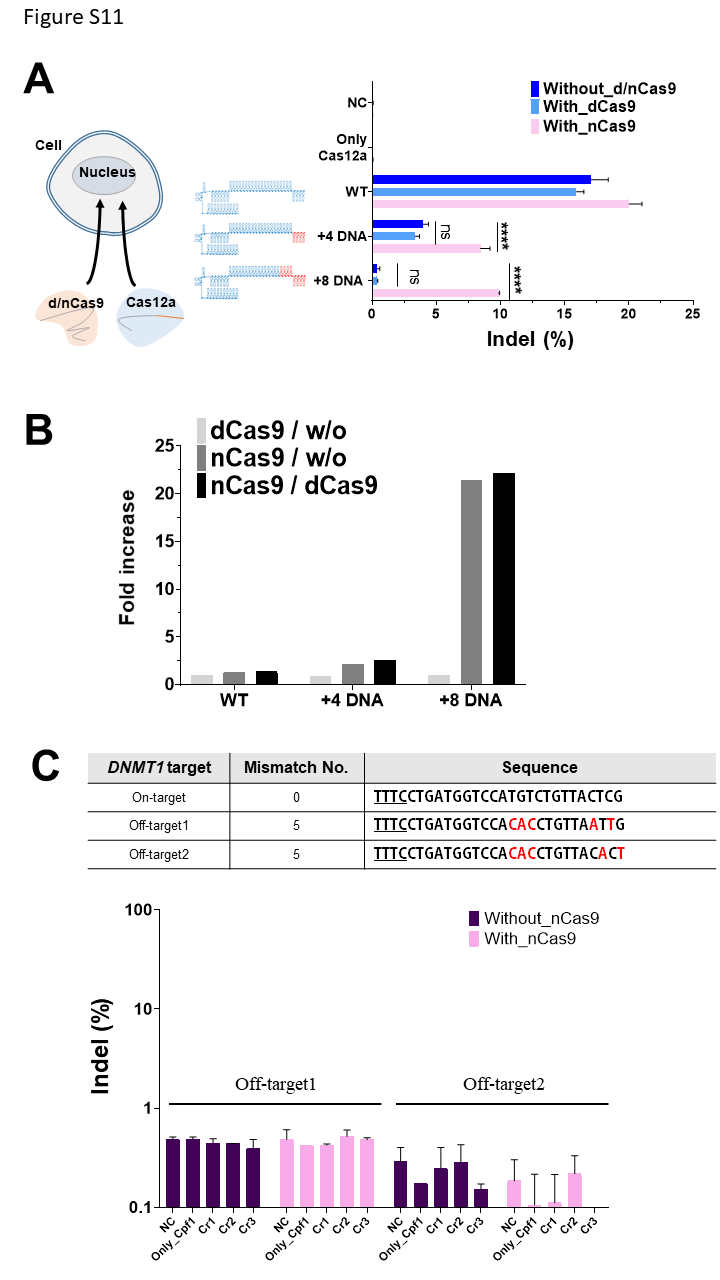


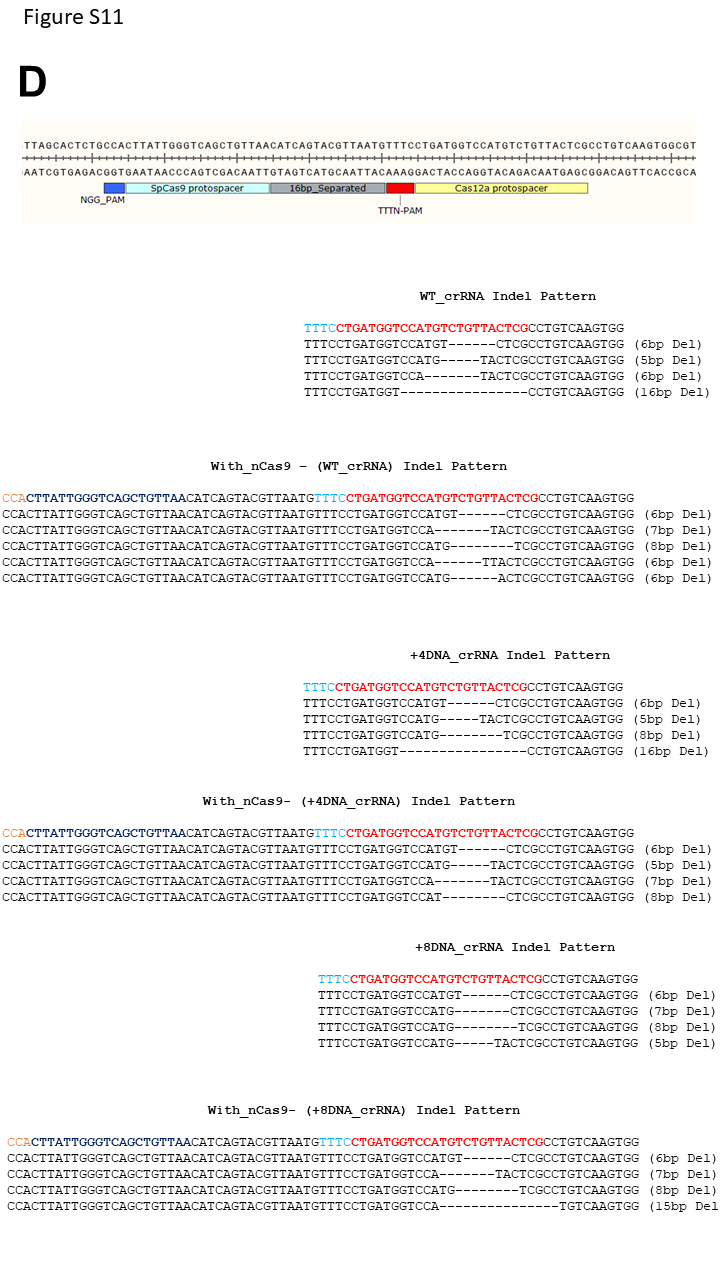


[Figure S11] The Cas12a editing efficiency and specificity are enhanced by combination of a chimeric DNA-RNA guide and SpCas9 nickase. (A) Intracellular *DNMT1* gene editing in HEK293FT cell using 3′-end DNA-substituted chimeric DNA-RNA-guided Cas12a and SpCas9 (D10A) nickase. The RNA portion of the Cas12a (cr)RNA is shown in blue, and the substituted DNA portion is shown in red. X-axis indicates the relative indel frequency (%). Only (cr)RNA - Cpf1 treated (dark blue) and combination with dCas9 (blue) or nCas9 (pink) treated samples are indicated, respectively. dCas9 and nCas9 indicate deactivated Cas9 (D10A, H840A) and nickase Cas9 (D10A), respectively. Indel ratio (%) is calculated by targeted amplicon sequencing from *DNMT1* site in HEK293FT cells (indel frequency (%) = mutant DNA read number / total DNA read number). Data are shown as means ± s.e.m. from three independent experiments. *P*-values are calculated using a two-tailed Student’s t-test (ns: not significant, P*:<0.05, P**:<0.01, P***:<0.001, P****:<0.0001). (B) Fold increase in the relative indel ratio (%) between only Cas12a treated, Cas12a and dCas9 co-treated, and Cas12a and nCas9 co-treated samples. Fold change is shown in light gray (dCas9 combination with Cas12a / only Cas12a treated), dark gray (nCas9 combination with Cas12a / only Cas12a treated) and black (nCas9 combination with Cas12a / dCas9 combination with Cas12a). (C) Off-target indel ratio (%) for site1 and site2 was calculated from targeted amplicon sequencing data in (A). Data are shown as means ± s.e.m. from three independent experiments. *P*-values are calculated using a two-tailed Student’s t-test (ns: not significant, P*:<0.05, P**:<0.01, P***:<0.001, P****:<0.0001). (D) Representative indel pattern from NGS analysis of chimeric (cr)RNA and nickase guided genome editing in HEK293FT cell. PAM sequence (TTTN) for AsCas12a in target sequence is shown in red and protospacer is shown in yellow. PAM sequence (NGG) for SpCas9 is shown in dark blue and protospacer is shown in light blue. Deleted sequence relative to the wild-type reference sequence is indicated by the dashed line. WT_crRNA: Wild-type crRNA was treated with Cas12a, +4 DNA_crRNA: Chimeric crRNA (sequential 4-nt DNA substitution at 3'-end of crRNA) was treated with Cas12a, +8 DNA_crRNA: Chimeric crRNA (sequential 8-nt DNA substitution at 3'-end of crRNA) was treated with Cas12a.


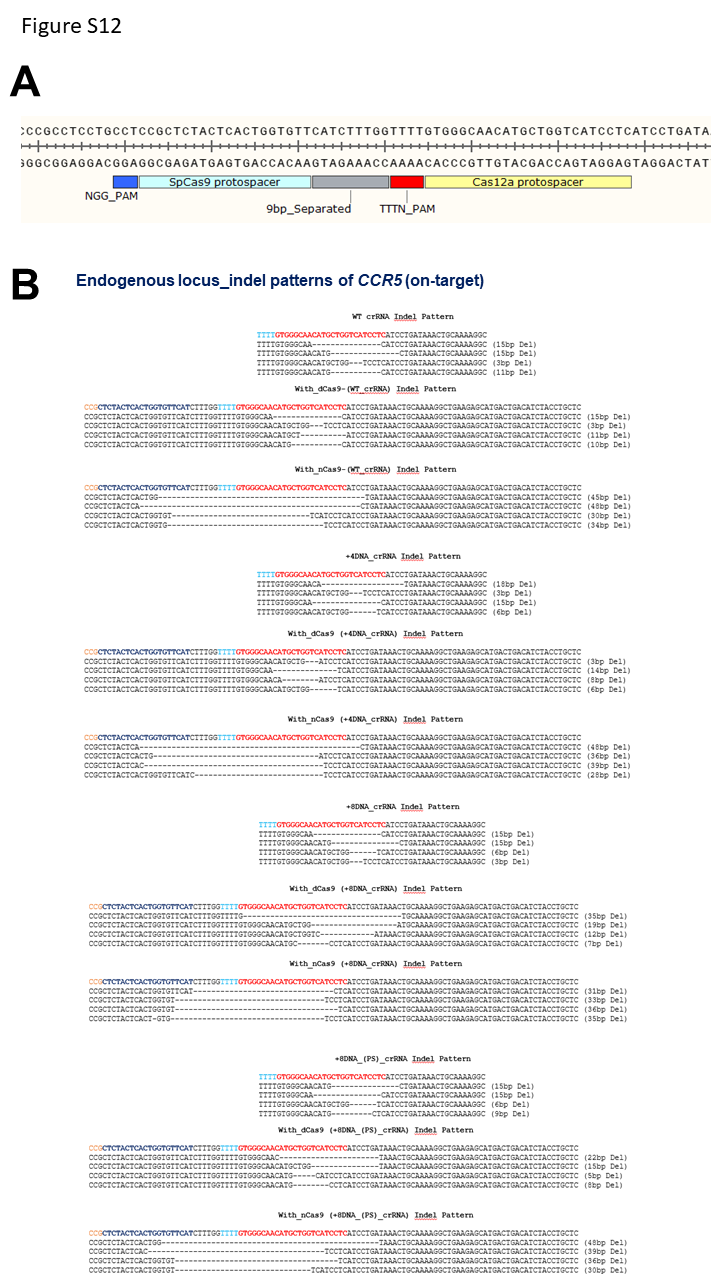


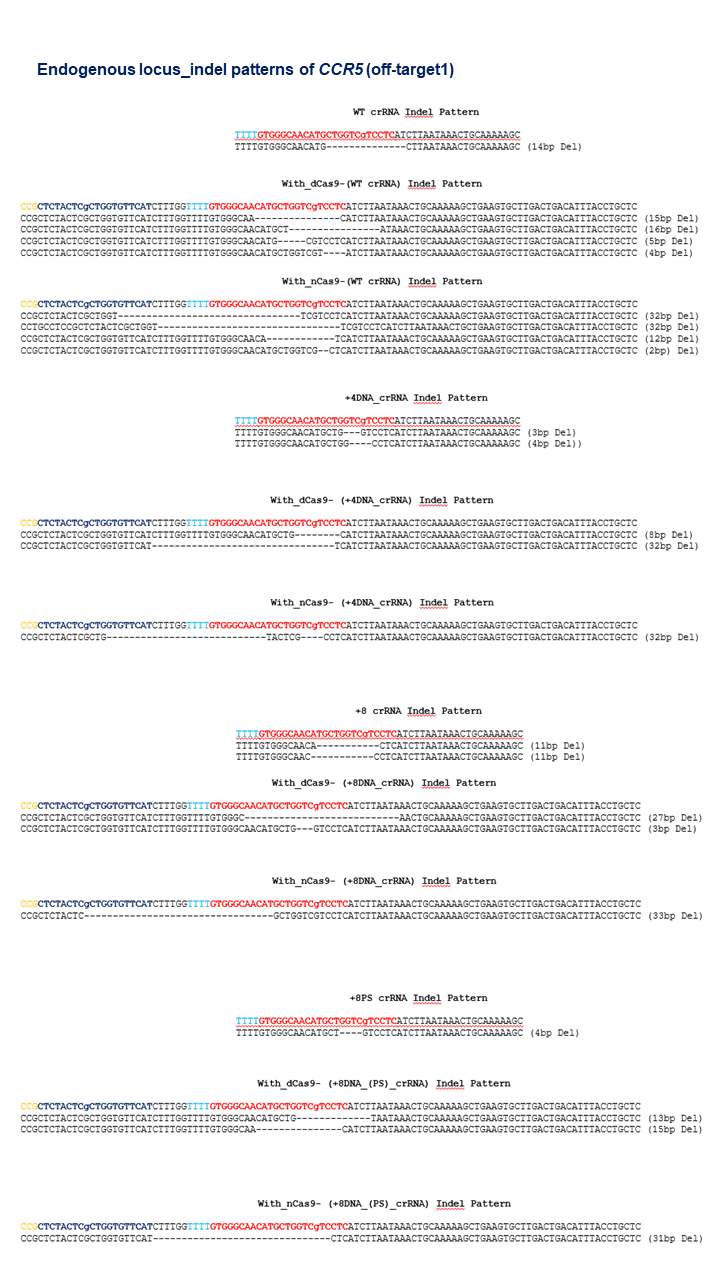


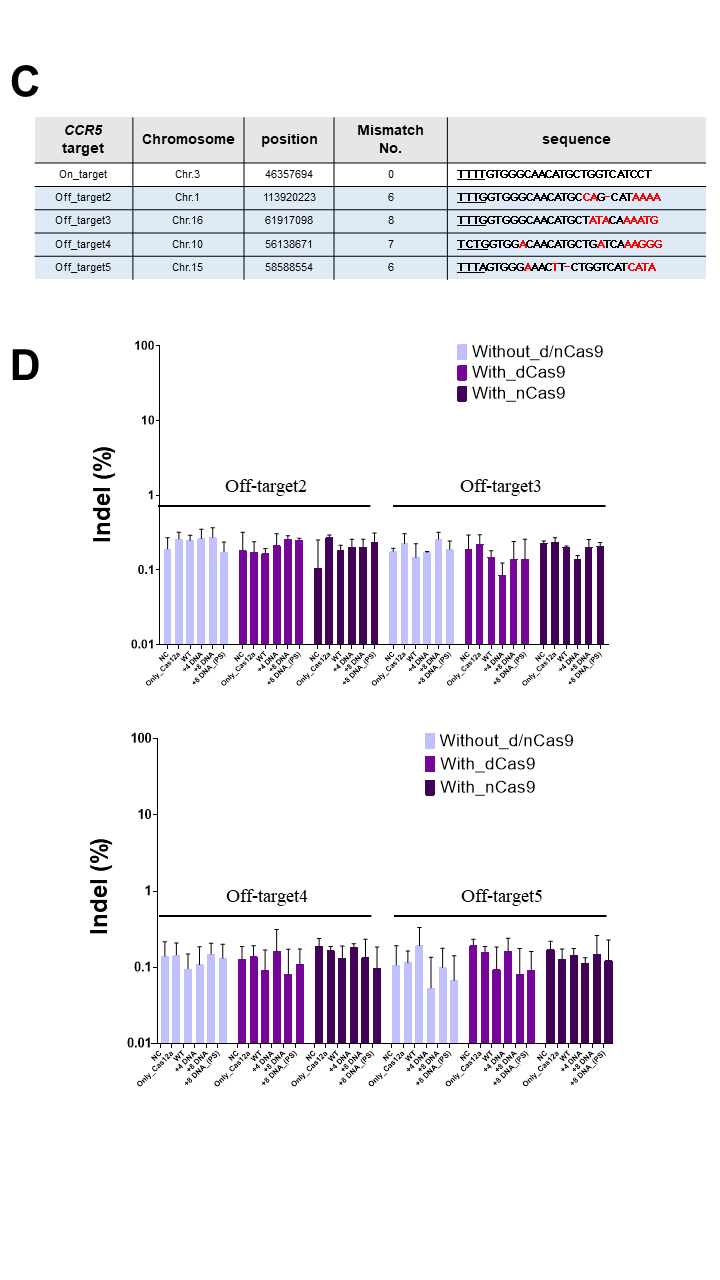


**[Figure S12] *CCR5* locus targeted genome editing with combination of d/n-SpCas9 and chimeric (cr)RNA guided AsCas12a (A)** Targeted endogenous locus of *CCR5* gene by AsCas12a and dead(D10A, H840A) or nickase (D10A) type of SpCas9 effectors. PAM sequences (NGG, TTTN) for SpCas9 and Cas12a effector are shown in red and blue, respectively and each target sequence is shown in yellow and light blue color. **(B)** Representative indel pattern from NGS analysis of chimeric (cr)RNA and d/n-SpCas9 guided genome editing in HEK293FT cell. PAM sequence (TTTN) for AsCas12a is shown in cyan and target sequence is shown in red. PAM sequence (NGG) for SpCas9 is shown in orange and target sequence is shown in dark blue. Deleted sequence relative to the wild-type reference sequence is indicated by the dashed line. WT_crRNA: Wild-type crRNA was treated with Cas12a, +4 DNA_crRNA: Chimeric crRNA (sequential 4-nt DNA substitution at 3'-end of crRNA) was treated with Cas12a, +8 DNA_crRNA: Chimeric crRNA (sequential 8-nt DNA substitution at 3'-end of crRNA) was treated with Cas12a. PS indicates phosphorothioate modification at 3’-end of crRNA. **(C)** *In-silico* predicted off-target candidates for *CCR5* on-target site. **(D)** Off-target indel ratio (%) for site2-5 was calculated from targeted amplicon sequencing data in (Figure7). Data are shown as means ± s.e.m. from three independent experiments. *P*-values are calculated using a two-tailed Student’s t-test (ns: not significant, P*:<0.05, P**:<0.01, P***:<0.001, P****:<0.0001).
